# Using PyBioNetFit to leverage qualitative and quantitative data in biological model parameterization and uncertainty quantification

**DOI:** 10.3389/fimmu.2026.1663008

**Published:** 2026-04-29

**Authors:** Ely F. Miller, Abhishek Mallela, Jacob Neumann, Yen Ting Lin, William S. Hlavacek, Richard G. Posner

**Affiliations:** 1 Department of Biological Sciences, Northern Arizona University, Flagstaff, AZ, United States; 2 Center for Nonlinear Studies, Los Alamos National Laboratory, Los Alamos, NM, United States; 3 Theoretical Biology and Biophysics Group, Theoretical Division, Los Alamos National Laboratory, Los Alamos, NM, United States; 4 Department of Chemistry and Chemical Biology, Cornell University, Ithaca, NY, United States; 5 Information Sciences Group, Computer, Computational and Statistical Sciences Division, Los Alamos National Laboratory, Los Alamos, NM, United States

**Keywords:** bayesian inference, curve-fitting, Markov chain Monte Carlo (MCMC), maximum likelihood estimation (MLE), profile likelihood

## Abstract

Data generated in studies of cellular regulatory systems are often qualitative. For example, measurements of signaling readouts in the presence and absence of mutations may reveal a rank ordering of responses across conditions but not the precise extents of mutation-induced differences. Qualitative data are often ignored by mathematical modelers or are considered in an *ad hoc* manner, as in the study of Kocieniewski and Lipniacki (2013) [*Phys Biol* 10: 035006], which was focused on the roles of MEK isoforms in ERK activation. In this earlier study, model parameter values were tuned manually to obtain consistency with a combination of qualitative and quantitative data. This approach is not reproducible, nor does it provide insights into parametric or prediction uncertainties. Here, starting from the same data and the same ordinary differential equation (ODE) model structure, we generate formalized statements of qualitative observations, making these observations more reusable, and we improve the model parameterization procedure by applying a systematic and automated approach enabled by the software package PyBioNetFit. We also demonstrate uncertainty quantification (UQ), which was absent in the original study. Our results show that PyBioNetFit enables qualitative data to be leveraged, together with quantitative data, in parameterization of systems biology models and facilitates UQ. These capabilities are important for reliable estimation of model parameters and model analyses in studies of cellular regulatory systems and reproducibility.

## 1 Introduction

In systems biology modeling, practitioners routinely disregard purely qualitative data—such as ordinal categorizations (e.g., low/medium/high), binary outcomes of case-control comparisons (e.g., up/down relative to a reference), and simple threshold crossing indicators (e.g., yes/no)—as well as semi-quantitative data, such as densitometric readouts of Western blots, which typically lack full quantification due to potential signal saturation and absence of calibration experiments. Bias against use of such data perhaps arises from the rich abundance of quantitative data (e.g., sequential measurements of state variables having high precision and fine time resolution) in fields such as physics, in which biological modelers are commonly trained. Nevertheless, qualitative/semi-quantitative data have been leveraged in influential biological modeling studies (1), such as those of Tyson and co-workers focused on modeling yeast cell-cycle control (2–6). In these studies, qualitative/narrative descriptions of yeast mutant phenotypes were used to constrain parameter estimates. Other related studies include those of Kinney and co-workers focused on using noisy semi-quantitative readouts of massively parallel reporter assays to inform the modeling of regulation of gene expression (7–10).

In recent years, there has been increasing appreciation of the need to leverage all types of available data in biological modeling, as evidenced by the development and/or application of various approaches for using qualitative/semi-quantitative data in model parameterization, optionally in combination with quantitative data. Demonstrated approaches include likelihood-free inference conditioned on binary data (11), data-transformation methods (e.g., optimal scaling) (12–16), information-theoretic approaches (9, 17), constrained heuristic optimization in which qualitative observations are formalized as constraints (18, 19), and (multinomial) probit regression (20). Software tools developed to cope with qualitative/semi-quantitative data include PyBioNetFit (20, 21), pyPESTO (15, 16, 22), and MAVE-NN (17).

Here, in a new demonstration of the ability of PyBioNetFit to leverage both quantitative and qualitative data, we redo the parameterization of an ordinary differential equation (ODE) model developed by Kocieniewski and Lipniacki (23) to study the roles of MEK isoforms in ERK activation. Originally, this model was parameterized by tedious trial-and-error manual tuning of parameter values for consistency with a mix of published qualitative and quantitative data (24, 25). The qualitative data included orderings of low-resolution readouts measured for parental and perturbed/mutant cell lines. In our re-analyses of these data, we define qualitative data unambiguously using the Biological Property Specification Language (BPSL) (21), which increases the reusability of these data, and we leverage both the qualitative and quantitative data using various features of PyBioNetFit, which yields a systematic and automated approach to parameter estimation and uncertainty quantification (UQ). This approach increases reproducibility. Furthermore, we clarify that the parameter estimation method of Mitra et al. (19), which is implemented in PyBioNetFit (21) and which we leveraged in our study, can be viewed as a likelihood-based inference procedure. Through metaheuristic optimization, we obtain maximum likelihood estimates for parameter values, and for UQ, we perform profile likelihood analysis (26). In addition, through application of an adaptive Markov chain Monte Carlo (MCMC) sampling algorithm (27) implemented in PyBioNetFit (28), we obtain samples representing a Bayesian parameter posterior, which allows us to quantify both parametric and prediction uncertainties. Prediction uncertainties are quantified by posterior predictive distributions. Thus, we demonstrate UQ in two ways, each of which considers both quantitative and qualitative data. The absence of UQ was a notable limitation of the original study of Kocieniewski and Lipniacki (23), a limitation rooted in the *ad hoc* labor-intensive parameterization approach of that study.

The results presented here provide a new illustration of how PyBioNetFit facilitates efficient and reproducible use of qualitative data (together with quantitative data) in parameterization of systems biology models. The results also provide an illustration of PyBioNetFit capabilities useful for UQ, which is essential for a variety of reasons, from assessment of model credibility to model improvement. This report should be useful to researchers who wish to leverage qualitative data in parameterization of biological models. For the convenience of readers unfamiliar with specialized terminology (e.g., BNGL, BPSL, UQ), we provide a glossary of key terms in the **Supplementary Material**.

## 2 Materials and methods

### 2.1 Model for parental cell line and model variants for mutant cell lines

Kocieniewski and Lipniacki (23) defined their model for normal, or wild type (WT), MEK1 signaling using the conventions of the BioNetGen language (BNGL) (29) and made the model available online in the form of a BNGL file (a plain-text file with a.bngl filename extension) within a ZIP archive (https://iopscience.iop.org/article/10.1088/1478-3975/10/3/035006/data). Annotation at the top of this BNGL file describes how to modify the model to account for MEK1 knockout (KO) and three MEK1 mutations: N78G, which ablates MEK1 dimerization with itself and MEK2; T292A, which ablates ERK-mediated inhibitory phosphorylation of MEK1 and concomitant negative feedback; and T292D, a phosphomimetic mutation. From this starting point, we created separate BNGL files for parental and mutant cell lines labeled WT, N78G, T292A, and T292D. The resulting BNGL files, MEK1_WT.bngl, MEK1_KO.bngl, MEK1_N78G.bngl, MEK1_T292A.bngl, and MEK1_T292D.bngl, are freely available online (https://github.com/lanl/PyBNF/tree/master/examples/Miller2025_MEK_Isoforms) and can be processed by PyBioNetFit (21), version 1.1.9. In PyBioNetFit workflows, models can be defined using either BNGL or Systems Biology Markup Language (SBML) (30). BioNetGen (31) can convert a BNGL file to an SBML file. SBML files are plain-text files with.xml filename extensions.

### 2.2 Data used in model parameterization

Kocieniewski and Lipniacki (23) carefully identified the data that they used in model parameterization. We collected these same data from the primary sources, namely the reports of Catalanotti et al. (24)Kamioka et al. (25). Each qualitative observation from the case-control comparisons of Catanotti et al. (24)—e.g., up/down relative to a reference at a particular time after initiation of signaling—was formalized using the Biological Property Specification Language (BPSL) (21). BPSL statements, which can be interpreted by PyBioNetFit, were then collected in PROP files (plain-text files with.prop filename extensions). See BPSL examples and breakdowns in the box below, and all BPSL statements used in our study are available in the **Supplementary Tables 1**–**5**. Quantitative observations, time-series data, from the study of Kamioka et al. (25) were collected/tabulated in an EXP file (a plain-text file with a.exp filename extension). It should be noted that, in an EXP file, “nan” indicates a missing measurement. The resulting EXP and PROP files are freely available online (https://github.com/lanl/PyBNF/tree/master/examples/Miller2025_MEK_Isoforms) and can be processed by PyBioNetFit (21), version 1.1.9. It should be noted that each EXP and PROP file is meant to be used with a particular model variant, as indicated by the labels WT, N78G, T292A, and T292D. For example, during model parameterization, the data collected in the WT-labeled EXP and PROP files (WT.exp and WT.prop) are compared against the corresponding outputs of the WT model (defined in the MEK1_WT.bngl file). The relevant model outputs are identified in accordance with established conventions (21); for example, in an EXP file, times are indicated by row labels and each column header has the name of an observable or function defined in a BNGL file. We note that the only non-empty EXP file maps to the WT model, because quantitative, time-series data was only available for normal signaling (i.e., signaling unperturbed by MEK1 knockout or mutation). Please see **Table 1** below.

**Table 1:** Examples of qualitative observations and their formal BPSL representations.

BPSL statement	Prose statement
WT.MEK_pRDS at time=300 < N78G.MEK_pRDS at time=300	The copy number of RAF-phosphorylated (pRDS) normal/wildtype (WT) MEK (in parental cells) is less than the copy number of RAF-phosphorylated MEK with mutation N78G (in a derived cell line) at 300 s after stimulation of RAS/RAF/MEK/ERK signaling.
N78G.pERK1_2_wt at time=300>T292D.pERK1_2_wt at time=300	The copy number of MEK1/2-double phosphorylated ERK with mutation N78G at 300 seconds is less than MEK-double phosphorylated ERK with mutation T292D at 300 seconds after simulation of RAS/RAF/MEK/ERK signaling.

### 2.3 Maximum likelihood estimation and likelihood profiling

Across the five model variants under consideration (WT, KO, N78G, T292A, and T292D), there are 46 total parameters (**Table 2**). We took 28 of these to be adjustable and set the other 18 at fixed values specified by Kocieniewski and Lipniacki (23). Six of these parameters ( $s_{1},\;d_{1},\;s_{2},\;d_{2},\;c_{1},\;c1_{init}$) correspond to the simulating the initial concentrations of EGFR, Sos1, and EGFR/ligand subcomplexes. In preliminary analyses, we found that estimating these parameters directly added unnecessary computational complexity without altering model behavior. Instead, we determined their peak concentrations from early simulation runs and fixed those values before initiating the cascade. This approach simplified parameterization while preserving biological realism. The remaining 12 fixed parameters $(X,MEK\_Fraction,\;EGFR_{0},\;SOS1_{0},\;RAS_{0},\;RAF_{0},\;MEK_{tot}0,\;MEK1_{0},\;MEK2_{0},\;$ $MEK1\_T292p_{0},\;ERK_{0},\;PHP\_MEK_{0})\;$define the initial conditions of the resting-state cell and mutants. These were held at the original values from Kocieniewski and Lipniacki (23) to maintain comparability with their published results.

**Table 2:** Original parameter value estimates of the MEK isoform model determined by Kocieniewski and Lipniacki (2013).

Parameter	Original parameter value	Parameter units	Parameter description
${\text{c}}_{1}\text{L}$	$\;2.0\text{x}10^{-2}\;$	Not Provided	c1 value after stimulation with ligand
${\text{c}}_{2}$	$2.0\text{x}10^{-7}$	${\left(mcls\;x\;s\right)}^{-1}$	EGFR receptor dimerization dueligand
${\text{t}}_{1}$	$1.0\text{x}10^{2}$	$s^{-1}$	EGFR subunits transphosphorylation in EGFR dimer
${\text{d}}_{3}$	$1.0\text{x}10^{-3}$	$s^{-1}$	degradation of ligand bound dimer complexes
${\text{b}}_{1}$	$4.0\text{x}10^{-8}$	${\left(mcls\;x\;s\right)}^{-1}$	association of phosphorylated receptor EGFR subunits with SOS1
${\text{n}}_{1}$	$2.0\text{x}10^{-3}$	$s^{-1}$	disassociation of EGFR receptor subunits from SOS1
${\text{b}}_{2}$	$1.0\text{x}10^{-5}$	${\left(mcls\;x\;s\right)}^{-1}$	MEK1 homodimer formation
${\text{n}}_{2}$	$1.0\text{x}10^{-3}$	$s^{-1}$	MEK1 dimer dissociation
${\text{b}}_{3}$	$1.0\text{x}10^{-5}$	${\left(mcls\;x\;s\right)}^{-1}$	MEK2 homodimer formation
${\text{n}}_{3}$	$3.0\text{x}10^{-2}$	$s^{-1}$	MEK2 homodimer dissociation
${\text{b}}_{4}$	$1.0\text{x}10^{-5}$	${\left(mcls\;x\;s\right)}^{-1}$	MEK1 and MEK2 heterodimer formation
${\text{n}}_{4}$	$1.0\text{x}10^{-3}$	$s^{-1}$	MEK1 and MEK2 heterodimer dissociation
${\text{a}}_{1}$	$1.5\text{x}10^{-7}$	${\left(mcls\;x\;s\right)}^{-1}$	activation of Ras by EGFR SOS1 complex (exchange of GDP for GTP)
${\text{i}}_{1}$	$2.0\text{x}10^{-2}$	$s^{-1}$	inactivation of RAS (hydrolysis of bound GTP to GDP)
${\text{a}}_{2}$	$4.0\text{x}10^{-8}$	${\left(mcls\;x\;s\right)}^{-1}$	activation of RAF by RAS-GTP
${\text{i}}_{2}$	$1.0\text{x}10^{-2}$	$s^{-1}$	inactivation of RAF
${\text{p}}_{1}$	$1.5\text{x}10^{-7}$	${\left(mcls\;x\;s\right)}^{-1}$	phosphorylation of MEK1 and MEK2 on the activation sites by RAF
${\text{u}}_{1}$	$5.0\text{x}10^{-3}$	$s^{-1}$	dephosphorylation of MEK1 and MEK2 on the activation sites
${\text{p}}_{2}\text{a}$	$1.0\text{x}10^{-6}$	${\left(mcls\;x\;s\right)}^{-1}$	phosphorylation of ERK on the activation sites by MEK1
${\text{p}}_{2}\text{b}$	$5.0\text{x}10^{-6}$	${\left(mcls\;x\;s\right)}^{-1}$	phosphorylation of ERK on the activation sites by MEK2
${\text{u}}_{2}$	$2.0\text{x}10^{-2}$	$s^{-1}$	dephosphorylation of ERK on the activation sites
${\text{p}}_{3}$	$2.0\text{x}10^{-9}$	${\left(mcls\;x\;s\right)}^{-1}$	feedback phosphorylation of SOS1 by active ERK
${\text{u}}_{3}$	$1.0\text{x}10^{-3}$	${\left(mcls\;x\;s\right)}^{-1}$	dephosphorylation of the SOS1 feedback site
${\text{p}}_{4}$	$1.2\text{x}10^{-9}$	${\left(mcls\;x\;s\right)}^{-1}$	feedback phosphorylation of MEK1 by ERK
${\text{u}}_{4}$	$2.0\text{x}10^{-4}$	${\left(mcls\;x\;s\right)}^{-1}$	dephosphorylation of the MEK1 feedback site (Thr292)
${\text{b}}_{5}$	$4.0\text{x}10^{-9}$	${\left(mcls\;x\;s\right)}^{-1}$	PHP phosphatse binding to Thr292p of MEK1
${\text{n}}_{5}$	$2.0\text{x}10^{-4}$	$s^{-1}$	dissociation of PHP phosphatase from Thr292p of MEK1
${\text{u}}_{5}$	$2.0\text{x}10^{1}$	$s^{-1}$	dephosphorylation of MEK1 and MEK2 on the activation sites
$s_{1}*$	2.5	$s^{-1}$	EGFR receptor subunit constitutive production
$d_{1}*$	$5.0\text{x}10^{-6}$	$s^{-1}$	EGFR receptor subunit constitutive degredation
$s_{2}*$	1.0	$s^{-1}$	Sos1 constitutive production
$d_{2}*$	$5.0\text{x}10^{-6}$	$s^{-1}$	Sos1 constitutive degredation
$c_{1}*$	$2.0\text{x}10^{-2}$	*N/A*	formation of EGFR receptor subunit-ligand complex Starting Signal
$c1_{init}*$	0.0	${\left(mcls\;x\;s\right)}^{-1}$	*c*_1_ value after simulation with ligand
MEK1_fraction*	$6.7\text{x}10^{-1}$	*N/A*	MEK1 fraction of total MEKs content
X*	5.0	*N/A*	MEK2 MEK1 kinase activity ratio
${\text{EGFR}}_{0}$*	$5.0\text{x}10^{5}$	*Copy Number*	initial EGFR level
$\text{SOS}1_{0}$*	$2.0\text{x}10^{5}$	*Copy Number*	initial Sos1 level
${\text{RAS}}_{0}$*	$5.0\text{x}10^{5}$	*Copy Number*	initial RAS level
${\text{RAF}}_{0}$*	$5.0\text{x}10^{5}$	*Copy Number*	initial RAF level
$\text{MEK}\_{\text{tot}}_{0}$*	$2.0\text{x}10^{5}$	*Copy Number*	initial MEK1 and MEK2 total level
$\text{MEK}1_{0}$*	$1.34\text{x}10^{5}$	*Copy Number*	initial MEK1 level
$\text{MEK}1\_\text{T}292{\text{p}}_{0}$*	0.0	*Copy Number*	initial MEK1-Thr292p level
$\text{MEK}2_{0}$*	$6.6\text{x}10^{4}$	*Copy Number*	initial MEK2 level
${\text{ERK}}_{0}$*	$3.0\text{x}10^{6}$	*Copy Number*	initial combined ERK1 and ERK2
$\text{PHP}\_{\text{MEK}}_{0}$*	$3.0\text{x}10^{6}$	*Copy Number*	level

All original 46 parameters are shown, including a brief description of their function within the MEK isoform model. Parameters marked with a single asterisk (*) denote parameters that were fixed during PyBioNetFit’s automated parameterization of the model.

Furthermore, all 28 adjustable parameters chosen to be used in our study were initially centered on Kocieniewski and Lipniacki’s (23) parameter values and given bounds of one or two orders of magnitude (10x - 100x) in either direction of the initial value (**Table 3**). Some parameter bounds were further adjusted if the algorithm began pushing the limit of a given bound. To ensure biological meaningfulness, we imposed physical constraints where applicable (e.g., rate constants bounded below by zero and above by the diffusion limit; fractional parameters bounded between 0 and 1). We made a conscious effort making these constraint choices to help maintain realism while supporting numerical stability in both optimization and Bayesian inference. All parameter bounds can be seen in **Table 3**. In addition, we introduced 3 adjustable scaling factors, which are useful for comparing WT model outputs to serial measurements. Consequently, in parameter estimation, we considered a total of 31 adjustable parameters, which we will denote as θ. To obtain point estimates, $\hat{\theta }$, for the 31 adjustable parameters θ, we minimized an objective function $F(\theta )=F_{\text{qual}}(\theta )+F_{\text{quant}}(\theta )$, the form of which was previously described by Mitra et al. (19). The objective function accounts for all qualitative data (a total of 90 BPSL statements) and all quantitative data (a total of 18 serial measurements). We constrained $\hat{\theta }$ to lie within a feasible region of parameter space, which we will denote as Θ. The feasible region Θ was defined by box constraints (lower and upper bounds) applied to each adjustable parameter. In summary, we found.

**Table 3:** Best-fit parameter estimates.

Parameter	Best-fit value	Optimization bounds [low, high]	Bayesian UQ bounds [low, high]
${\text{c}}_{1}\text{L}$	$7.4\text{x}10^{-3}$	$\left[9.0\text{x}10^{-4},\;4.0\text{x}10^{-2}\right]$	N/A
${\text{c}}_{2}$	$9.3\text{x}10^{-9}$	$\left[9.0\text{x}10^{-9},\;4.0\text{x}10^{-6}\right]$	N/A
${\text{t}}_{1}$	$9.9\text{x}10^{1}$	[99, 101]	N/A
${\text{d}}_{3}$	$2.0\text{x}10^{-3}$	$\left[8.0\text{x}10^{-5},\;3.0\text{x}10^{-2}\right]$	$\left[1.0\text{x}10^{-5},\;1.0\text{x}10^{-1}\right]$
${\text{b}}_{1}$	$2.4\text{x}10^{-8}$	$\left[2.0\text{x}10^{-9},\;6.0\text{x}10^{-7}\right]$	N/A
${\text{n}}_{1}$	$6.5\text{x}10^{-4}$	$\left[9.0\text{x}10^{-5},\;4.0\text{x}10^{-2}\right]$	N/A
${\text{b}}_{2}$	$4.2\text{x}10^{-6}$	$\left[8.0\text{x}10^{-7},\;3.0\text{x}10^{-4}\right]$	N/A
${\text{n}}_{2}$	$2.6\text{x}10^{-4}$	$\left[8.0\text{x}10^{-5},\;3.0\text{x}10^{-2}\right]$	N/A
${\text{b}}_{3}$	$9.9\text{x}10^{-5}$	$\left[8.0\text{x}10^{-7},\;3.0\text{x}10^{-4}\right]$	N/A
${\text{n}}_{3}$	$8.0\text{x}10^{-2}$	$\left[1.0\text{x}10^{-3},\;5.0\text{x}10^{-1}\right]$	N/A
${\text{b}}_{4}$	$2.9\text{x}10^{-4}$	$\left[8.0\text{x}10^{-7},\;3.0\text{x}10^{-4}\right]$	N/A
${\text{n}}_{4}$	$3.5\text{x}10^{-3}$	$\left[8.0\text{x}10^{-5},\;3.0\text{x}10^{-2}\right]$	N/A
${\text{a}}_{1}$	$2.3\text{x}10^{-7}$	$\left[1.27\text{x}10^{-8},\;1.29\text{x}10^{-6}\right]$	N/A
${\text{i}}_{1}$	$3.9\text{x}10^{-1}$	$\left[9.0\text{x}10^{-4},\;4.0\text{x}10^{-1}\right]$	N/A
${\text{a}}_{2}$	$3.3\text{x}10^{-8}$	$\left[2.0\text{x}10^{-9},\;6.0\text{x}10^{-7}\right]$	N/A
${\text{i}}_{2}$	$2.7\text{x}10^{-2}$	$\left[8.0\text{x}10^{-4},\;3.0\text{x}10^{-1}\right]$	N/A
${\text{p}}_{1}$	$3.5\text{x}10^{-6}$	$\left[8.0\text{x}10^{-9},\;4.0\text{x}10^{-6}\right]$	N/A
${\text{u}}_{1}$	$9.5\text{x}10^{-4}$	$\left[3.0\text{x}10^{-4},\;7.0\text{x}10^{-2}\right]$	N/A
${\text{p}}_{2}\text{a}$	$5.2\text{x}10^{-7}$	$\left[8.0\text{x}10^{-8},\;3.0\text{x}10^{-5}\right]$	N/A
${\text{p}}_{2}\text{b}$	$2.3\text{x}10^{-5}$	$\left[1.0\text{x}10^{-6},\;5.0\text{x}10^{-4}\right]$	N/A
${\text{u}}_{2}$	$6.7\text{x}10^{-2}$	$\left[9.0\text{x}10^{-4},\;4.0\text{x}10^{-1}\right]$	N/A
${\text{p}}_{3}$	$1.1\text{x}10^{-9}$	$\left[9.0\text{x}10^{-11},\;4.0\text{x}10^{-8}\right]$	N/A
${\text{u}}_{3}$	$2.6\text{x}10^{-4}$	$\left[8.0\text{x}10^{-5},\;3.0\text{x}10^{-2}\right]$	$\left[1.0\text{x}10^{-5},\;1.0\text{x}10^{-1}\right]$
${\text{p}}_{4}$	$6.5\text{x}10^{-10}$	$\left[9.0\text{x}10^{-11},\;3.0\text{x}10^{-8}\right]$	N/A
${\text{u}}_{4}$	$3.2\text{x}10^{-4}$	$\left[9.0\text{x}10^{-6},\;4.0\text{x}10^{-3}\right]$	N/A
${\text{b}}_{5}$	$1.7\text{x}10^{-8}$	$\left[2.0\text{x}10^{-10},\;6.0\text{x}10^{-8}\right]$	N/A
${\text{n}}_{5}$	$3.9\text{x}10^{-3}$	$\left[9.0\text{x}10^{-6},\;4.0\text{x}10^{-3}\right]$	N/A
${\text{u}}_{5}$	$1.9\text{x}10^{1}$	[19, 21]	N/A
${\text{Scale}}_{\text{pEGFR}}$*	$1.1\text{x}10^{-4}$	$\left[3.0\text{x}10^{-5},\;6.0\text{x}10^{-5}\right]$	$\left[3.0\text{x}10^{-5},\;6.0\text{x}10^{-5}\right]$
${\text{Scale}}_{\text{pERK}}$*	$3.2\text{x}10^{-6}$	$\left[2.0\text{x}10^{-6},\;5.0\text{x}10^{-6}\right]$	$\left[2.0\text{x}10^{-6},\;5.0\text{x}10^{-6}\right]$
${\text{Scale}}_{\text{pSOS}1}$*	$1.1\text{x}10^{-4}$	$\left[9.0\text{x}10^{-5},\;3.0^{-4}\right]$	$\left[9.0\text{x}10^{-5},\;3.0^{-4}\right]$
sigma**	1.1	N/A	$\left[1.0\text{x}10^{-1},\;1.0\text{x}10^{1}\right]$

This table lists the 31 parameters selected for model calibration using PyBioNetFit. Each row indicates the name of a parameter, the best-fit value obtained from a global fit, and parameter bounds used in optimization and Bayesian UQ (if applicable). Parameters marked with a single asterisk (*) are scaling factors added specifically for the WT model to allow model outputs to be compared to published measurements reported in arbitrary units (AU). Parameters with a double asterisk (**) are special hyper sampling parameters used only in Bayesian UQ simulations needed for PyBioNetFit’s Adaptive MCMC algorithm and not originally included in the model by Kocieniewski and Lipniacki (2013).

(1) $$\hat{\theta }=\arg\underset{\theta \in \text{Θ}}{\min}F(\theta )$$

Thus, $\hat{\theta }$ is the product of a global fit. As explained below, the objective function F(θ) is related to a likelihood, and moreover, minimizing the objective function maximizes this likelihood. Thus, our point estimates are maximum likelihood estimates (MLEs).

The fitting problem setup, including identification of each adjustable parameter and the corresponding box constraints, and workflow were defined with a PyBioNetFit configuration, or CONF, file. This file (a plain-text file with a.conf filename extension) is freely available online (https://github.com/lanl/PyBNF/tree/master/examples/Miller2025_MEK_Isoforms/MEK_isoform_optimization_DE). In parameter estimation, the objective function F(θ) was minimized using PyBioNetFit’s implementation of a parallelized differential evolution (DE) algorithm (21). PyBioNetFit-enabled fitting runs were executed within an institutional high-performance computing environment, on the Monsoon cluster at Northern Arizona University. We used 25 of 28 CPUs on a single node within the cluster and the average wall-clock time for optimization simulations were approximately 2 minutes and conducted 2,825 objective function evaluations. The model of CPUs used are Intel(R) Xeon(R) CPU E5–2680 v4 CPUs. Multiple runs were performed, with each run starting at a randomly chosen point within the feasible region of parameter space Θ. The CONF file includes algorithmic parameter settings (e.g., population size) and maps EXP and PROP files to model variants.

Profile likelihood analysis (26) was performed as described by Mitra et al. (19). Computation of a profile involves repeatedly solving the parameterization problem described above, but with one selected parameter in θ held fixed at a specified value, which is varied across the profile. This analysis is available in the **Supplementary Material** as **Supplementary Figure 4**. We found that 14 of the 28 parameters selected for maximum likelihood estimation were identifiable, while the remaining parameters showed little sensitivity to perturbation from their original values.

When included in maximum likelihood estimation (MLE), unidentifiable parameters can reduce the identifiability of sensitive parameters if they are not fixed during parameterization. To assess whether estimating all 28 parameters affected PyBioNetFit’s parameterization of the MEK isoform models, we performed two fits. In the first, MLEs were estimated for all 28 parameters. In the second, only the 14 identifiable parameters were estimated, while the remaining were fixed to the original values from Kocieniewski and Lipniacki (23). Using PyBioNetFit’s differential evolution algorithm and sum of squares objective function, we found that fixing the non-sensitive parameters did not significantly alter the overall objective score or the estimates of identifiable parameters.

### 2.4 Bayesian inference and uncertainty quantification

In Bayesian inference and uncertainty quantification, for simplicity, we considered only two adjustable model parameters: $d_{3}$, a rate constant characterizing degradation of ligand-bound epidermal growth factor receptor (EGFR) dimers, and $u_{3}$, a rate constant characterizing phosphatase activity responsible for reversal of ERK-mediated negative-feedback phosphorylation of SOS1. Both parameters were deemed practically identifiable by profile likelihood. The adjustable parameters also included three scaling factors, as in fitting. Finally, the adjustable parameters included a noise model parameter, σ. In Bayesian inference, we used a likelihood function described by Mitra and Hlavacek (20), which has σ as a hyperparameter, and a proper uniform prior defined by box constraints on each of the six adjustable parameters (see below).

Bayesian inference was enabled by the adaptive Markov chain Monte Carlo (MCMC) sampler implemented in PyBioNetFit (28), version 1.1.9. The sampling problem setup and workflow were defined with a PyBioNetFit configuration, or CONF file. This file (a plain-text file with a.conf filename extension) is freely available online (https://github.com/lanl/PyBNF/tree/master/examples/Miller2025_MEK_Isoforms/MEK_isoform_aMCMC). The CONF file specifies the box constraints that define the prior. Each sampling job included 25,000 burn-in iterations, 25,000 adaptation iterations (used to tune the covariance matrix of the proposal kernel), and 250,000 production iterations. We generated five independent chains. Each chain was initialized at a random point within the prior. All five chains converged to the same posterior distribution. Convergence was evaluated by inspecting diagnostic trace plots and pairs plots. We also calculated convergence metrics (32) using the rstan R package (**Table 4**). These metrics indicated convergence according to the guidance of Vehtari et al. (32). PyBioNetFit-enabled sampling jobs were executed within an institutional high-performance computing environment, on the Monsoon cluster at Northern Arizona University. In the Monsoon cluster at Northern Arizona University, we used 25 CPUs of a single 28 CPU node containing Intel(R) Xeon(R) CPU E5–2680 v4 CPUs, and the approximate wall-clock time for our Bayesian inference simulations were 5.15 days, having conducted 1.25 million objective function evaluations.

### 2.5 Derivation of likelihood function

In this study, we consider a set of model variants M and a dataset D={y,z}. The dataset consists of n quantitative observations, $y=\{y_{1},\ldots ,y_{n}\}$, and *m* qualitative observations, $z=\{z_{n+1},\ldots ,z_{n+m}\}$. As a simplification, we assume that the n+m observations are independent.

The quantitative observations y are relative intensity measurements, which have been scaled such that $y_{i}\in (0,1]$ for i=1,…,n. There are no replicate measurements, and each $y_{i}$ corresponds to a unique measurement condition, $c_{i}$. For each experimental readout $y_{i}$, there is a corresponding model output $f(c_{i},\text{θ})$, which depends on condition $c_{i}$ and P adjustable parameters, $\text{θ}=\{{\text{θ}}_{1},\ldots ,{\text{θ}}_{P}\}$. The model output $f(c_{i},\text{θ})$ is generated by the model variant in M matched to condition $c_{i}$, which is always the WT model (because time-series data are not available for any of the mutant cells).

Each qualitative observation $z_{i}\in \{0,1\}$ ( i=n+1,…,n+m) is the binary outcome of a comparison of two semi-quantitative measurements $A_{i}$ and $B_{i}$ made for two different cell lines (e.g., parental and mutant cells) or at two different time points within the same cellular background. By convention, we take $z_{i}=0$ to indicate $A_{i}<B_{i}$ and $z_{i}=1$ to indicate $A_{i}\geq B_{i}$. The measurements $A_{i}$ and $B_{i}$ are made at conditions $a_{i}$ and $b_{i}$, which are identical except for the difference in cell type or time. For each pair of measurements $\left(A_{i},B_{i}\right)$, there is a pair of model outputs $\left(g(a_{i},\theta ),\;g(b_{i},\theta )\right)$. The output $g(a_{i},\theta )$ is generated by the model variant in M matched to condition $a_{i}$, which encompasses cell type or time, and similarly, the output $g(b_{i},\theta )$ is generated by the model variant matched to $b_{i}$. Moreover, for each $z_{i}$, we have a corresponding model prediction $H(g(a_{i},\;\theta )-g(b_{i},\theta ))$, where H is the Heaviside function.

In maximum likelihood estimation, we want to find $\hat{\text{θ}}={\text{argmax}}_{\text{θ}\in \text{Θ}}L(\text{θ|}D)$, where the likelihood L(θ|D)=P(D|θ) is the probability density over the data D given model structure and parameter settings. In inference, we view L(θ|D) as a function of the adjustable parameters θ and take L(θ|D) to express how probable the data are for given parameter settings. If observations are independent, L(θ|D)=L(θ|{y,z}) decomposes into the product L(θ|y)L(θ|z), where L(θ|y) is the likelihood of the quantitative data and L(θ|z) is the likelihood of the qualitative data.

Let us use $Y_{i}$ to denote a continuous random variable representing the distribution of possible quantitative measurement outcomes of an experiment performed at condition $c_{i}$. We take the observation $y_{i}$ to be a realization of $Y_{i}$, and we take the model output $f(c_{i},\text{θ})$ to be a prediction of the expected measurement outcome at condition $c_{i}$. Formally, we take $f(c_{i},\text{θ})$ to model $E[Y_{i}]$. Let us assume normally distributed measurement noise: $Y_{i}∼N(f(c_{i},\text{θ}),{\text{σ}}_{i}^{2})$. It then follows that.

(2) $$P(Y_{i}=y_{i}|f(c_{i},\text{θ}))=\frac{1}{{\text{σ}}_{i}\sqrt{2\text{π}}}\exp[-\frac{1}{2}{\left(\frac{y_{i}-f(c_{i},\text{θ})}{{\text{σ}}_{i}}\right)}^{2}]$$

If the quantitative data $y={\left\{y_{i}\right\}}_{i=1}^{n}$ are taken to be independent, we find.

(3) $$P(y|{\left\{f(c_{i},\text{θ})\right\}}_{i=1}^{n})=\prod_{i=1}^{n}\;\frac{1}{{\text{σ}}_{i}\sqrt{2\text{π}}}\exp[-\frac{1}{2}{\left(\frac{y_{i}-f(c_{i},\text{θ})}{{\text{σ}}_{i}}\right)}^{2}]$$

We can identify $P(y|{\left\{f(c_{i},\text{θ})\right\}}_{i=1}^{n})$ as L(θ|y). In the absence of information about variances, let us assume that ${\text{σ}}_{i}^{2}={\text{σ}}^{2}>0$ for i=1,…,n. Under this assumption of homoscedasticity (i.e., equal variances), we find (Equations 4, 6–10).

(4) $$\;\;-\ln L(\text{θ|}y)=\frac{n}{2}\ln(2{\text{πσ}}^{2})+\frac{1}{2{\text{σ}}^{2}}\sum_{i=1}^{n}{\left(y_{i}-f(c_{i},\text{θ})\right)}^{2}$$

Furthermore,

(5) $$\;\;-\ln L(\text{θ|}y)\propto F_{\text{quant}}\equiv \sum_{i=1}^{n}{\left(y_{i}-f(c_{i},\text{θ})\right)}^{2}$$

Note that L(θ|y) is maximized if we minimize $F_{\text{quant}}$. The expression for $F_{\text{quant}}$ is equivalent to Equation 2 in Mitra et al. (19).

Let us use $Z_{i},i\in \{n+1,\ldots ,m+n\}$, to denote a discrete random variable representing the distribution of possible outcomes from a comparison of two semi-quantitative experimental readouts $A_{i}$ and $B_{i}$ made at conditions $a_{i}$ and $b_{i}$. There are only two possible outcomes, which are mutually exclusive: $Z_{i}=0$ (indicating that $A_{i}<B_{i}$) or $Z_{i}=1$ (indicating that $A_{i}\geq B_{i}$). We take $z_{i}$ to be a realization of $Z_{i}$. We will assume the following noise model: $Z_{i}∼\text{Bernoulli}(p_{i})$. Equivalently,

(6) $$\text{Pr}(Z_{i}=0)=1-p_{i}\text{ and Pr}(Z_{i}=1)=p_{i}$$

Note that $p_{i}$ is the expectation $E[Z_{i}]$. Recall that the model prediction of $z_{i}$ is $H(g(a_{i},\;\theta )-g(b_{i},\theta ))$. As in standard logistic regression, we will assume that the parameter $p_{i}$ is a sigmoid function of the difference ${\text{δ}}_{i}\equiv g(a_{i},\text{θ})-g(b_{i},\text{θ})$:

(7) $$p_{i}=\frac{1}{1+\exp[-{\text{δ}}_{i}/s_{i}]}$$

where $s_{i}>0$ is a scale parameter. With this approach, we are assuming that the difference $\delta_{i}$ explains the probability $p_{i}$. If ${\text{δ}}_{i}\geq 0$, then $p_{i}$ lies between 0.5 and 1 and $z_{i}=1$ is more likely than $z_{i}=0$. Conversely, if ${\text{δ}}_{i}<0$, then $p_{i}$ lies between 0 and 0.5 and $z_{i}=1$ is less likely than $z_{i}=0$. From the above considerations, we find.

(8) $$P(Z_{i}=z_{i}{\text{|δ}}_{i},s_{i})={\left(\frac{1}{1+e^{-{\text{δ}}_{i}/s_{i}}}\right)}^{z_{i}}{\left(\frac{e^{-{\text{δ}}_{i}/s_{i}}}{1+e^{-{\text{δ}}_{i}/s_{i}}}\right)}^{1-z_{i}}$$

If the data $z={\left\{z_{i}\right\}}_{i=n+1}^{n+m}$ are independent, we find.

(9) $$P(z|{\left\{{\text{δ}}_{i}\right\}}_{i=n+1}^{n+m},{\left\{s_{i}\right\}}_{i=n+1}^{n+m})=\prod_{i=n+1}^{n+m}\;{\left(\frac{1}{1+e^{-{\text{δ}}_{i}/s_{i}}}\right)}^{z_{i}}{\left(\frac{e^{-{\text{δ}}_{i}/s_{i}}}{1+e^{-{\text{δ}}_{i}/s_{i}}}\right)}^{1-z_{i}}$$

We can identify $P(z|{\left\{{\text{δ}}_{i}\right\}}_{i=n+1}^{n+m},{\left\{{\text{s}}_{i}\right\}}_{i=n+1}^{n+m})$ as L(θ|z). After simplifications, we find.

(10) $$-\ln L(\text{θ|}z)=\sum_{i=n+1}^{n+m}\left[\ln(1+e^{-{\text{δ}}_{i}/s_{i}})+(1-z_{i}){\text{δ}}_{i}/s_{i}\right]$$

Furthermore, under an assumption that $|\frac{\delta_{i}}{s_{i}}|\gg 1$ for i=n+1,…,n+m, we find.

(11) $$-\ln L(\text{θ|}z)\approx F_{\text{qual}}\equiv \sum_{i=n+1}^{n+m}w_{i}[\max(0,-{\text{δ}}_{i})+(1-z_{i}){\text{δ}}_{i}]$$

where $w_{i}=1/s_{i}$. The ith term in the sum can be viewed as a static penalty function with weight $w_{i}$. The penalty is $w_{i}\cdot |{\text{δ}}_{i}|$ if the explanatory variable ${\text{δ}}_{i}$ is inconsistent with the observation $z_{i}$ and 0 otherwise. In the absence of information indicating how categorizations vary over a range of values for the explanatory variable, the weights can be set heuristically as described by Mitra et al. (19). The above expression for $F_{\text{qual}}$ is equivalent to Equation 3 in Mitra et al. (19).

In Equation 1, F(θ) is equal to $F_{\text{quant}}(\theta )+F_{\text{qual}}(\theta )$, where $F_{\text{quant}}(\theta )$ is given by Equation 5 and $F_{\text{qual}}(\theta )$ is given by Equation 11.

## 3 Results

Kocieniewski and Lipniacki (23) developed a collection of related ordinary differential equation (ODE) models for ERK activation dynamics. These models capture and explain MEK isoform-specific effects in cell lines in which MEK1 is normally expressed (WT), knocked out (KO), and mutated at single amino-acid residues (N78G, T292A, and T292D). The models reproduce experimentally characterized signaling behavior, including various qualitative system properties; however, their parameter values were determined through an *ad hoc* manual trial-and-error procedure, precluding straightforward reuse of the qualitative data (up/down assays relative to a reference), reproducibility of the model parameterization approach, and any formal assessment of parametric and prediction uncertainty.

**Figure 1:**
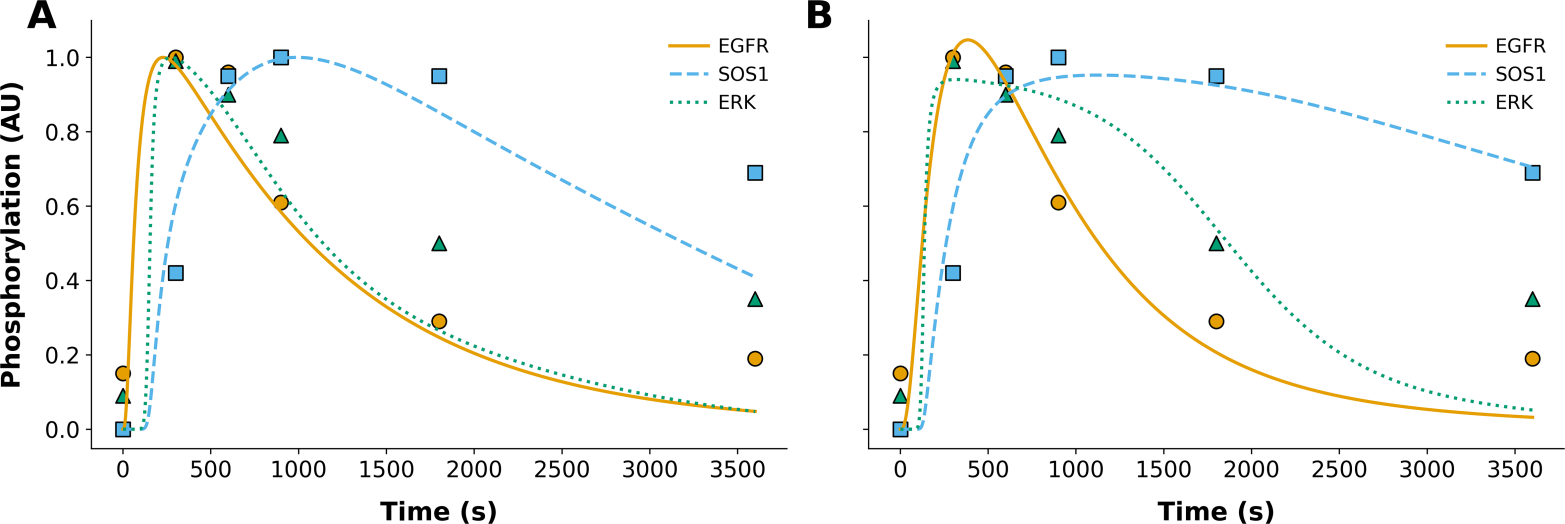
Comparison of model-generated best-fit trajectories with experimental phosphorylation data in WT conditions. Each panel displays simulated trajectories for phosphorylated EGFR (solid orange), SOS1 (dashed light blue), and ERK (dotted green), plotted alongside their respective experimental data points (colored to match model predictions). All trajectories are based on MLEs for parameters. Panel **(A)** shows the model outputs derived from the original parameterization of Kocieniewski and Lipniacki (2013). Panel **(B)** presents the MLEs obtained using PyBioNetFit, as described in Materials and Methods. Phosphorylation values are reported in arbitrary units (AU). Experimental data points are consistent between panels.

**Figure 2:**
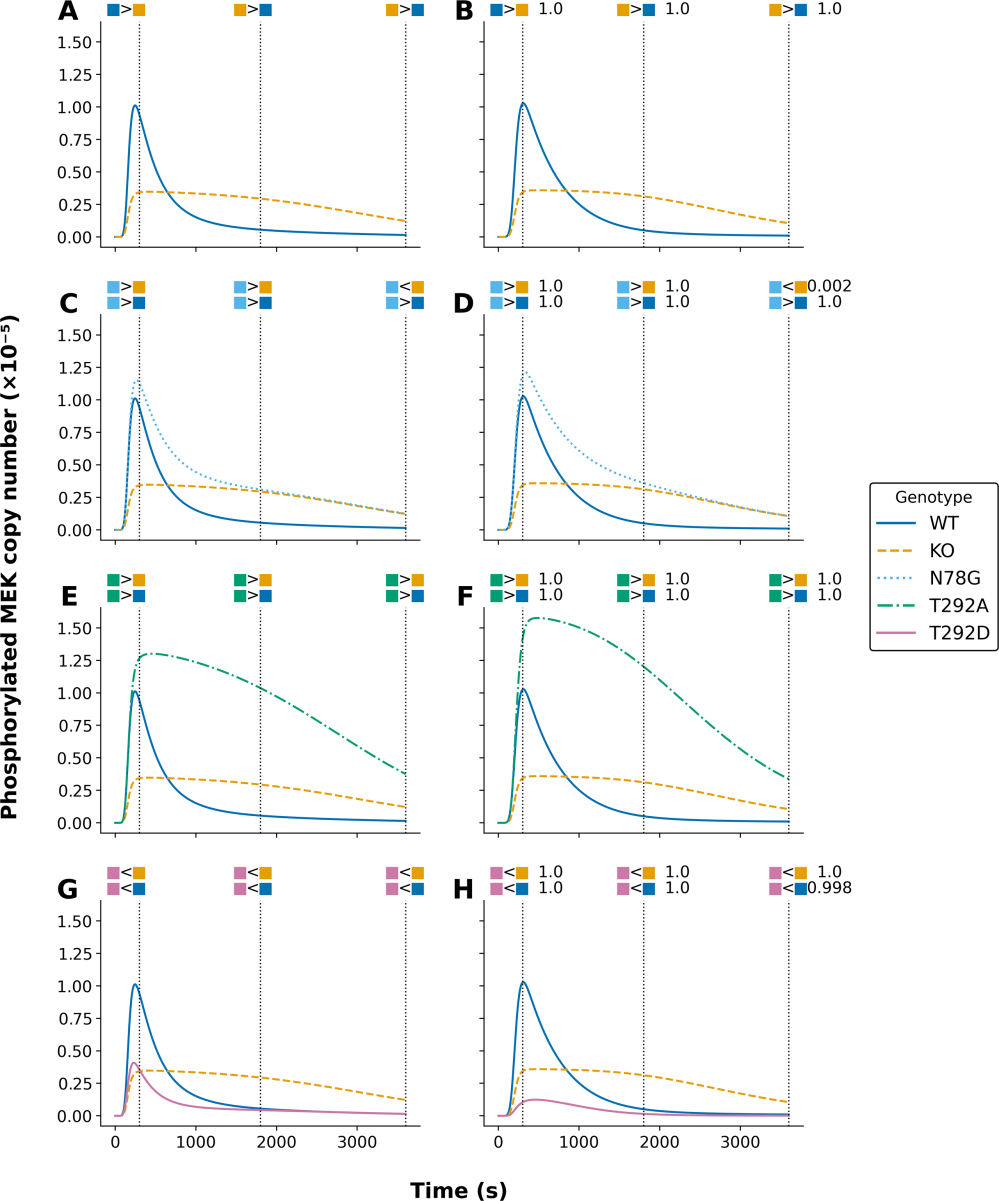
Comparison of model-predicted phosphorylated MEK trajectories under different parameterizations for five model variants. Each panel illustrates trajectories for phosphorylated MEK (in molecules ×10^-5^) for indicated models. Curves are color-coded by model variant. wild type (WT, solid blue), knockout (KO, dashed orange), N78G (dotted light blue), T292A (dashed-dotted green), and T292D (solid pink). The left panels **(A, C, E, G)** display model outputs derived from the original parameterization of Kocieniewski and Lipniacki (2013). The right panels **(B, D, F, H)** show outputs based on the parameterization obtained using PyBioNetFit, as described in Materials and Methods. Constraints used in parameterization are represented as colored glyphs above each panel and match the corresponding model variant by color. Vertical black dotted lines indicate the time points at which these constraints apply. 300, 1800, and 3600 seconds. For **(B, D, F, H)** (PyBioNetFit parameterization), numerical annotations indicate the fraction of accepted MCMC samples satisfying each constraint.

**Figure 3:**
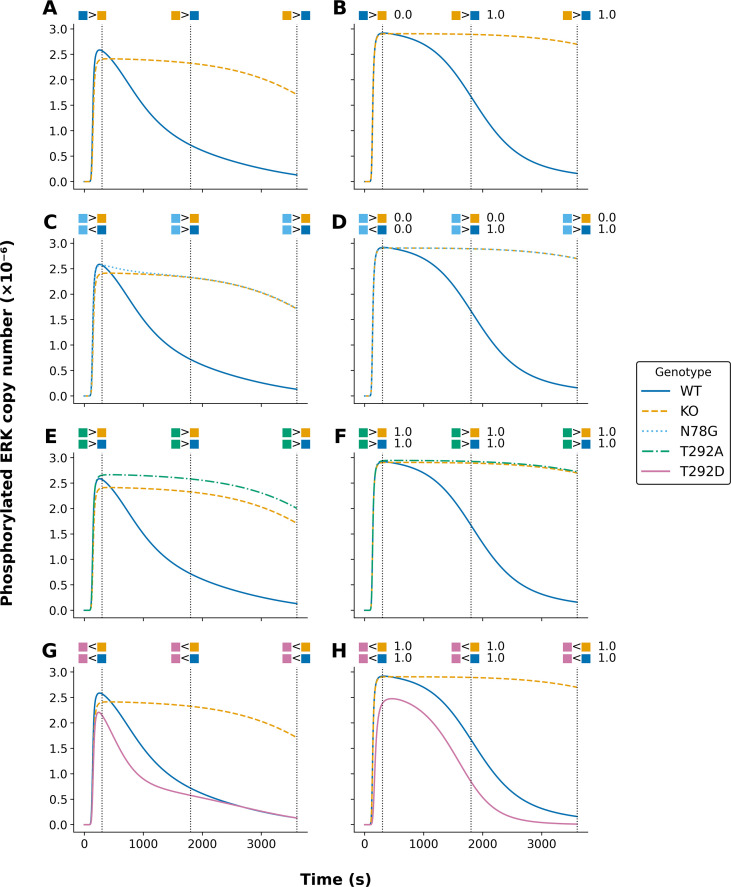
Comparison of model-predicted phosphorylated ERK trajectories under different parameterizations for five model variants. Each panel illustrates trajectories for phosphorylated ERK (in molecules ×10^-6^) for indicated models. Curves are color-coded by model variant: wild type (WT, solid blue), knockout (KO, dashed orange), N78G (dotted light blue), T292A (dashed-dotted green), and T292D (solid pink). The left panels **(A, C, E, G)** display model outputs derived from the original parameterization of Kocieniewski and Lipniacki (2013). The right panels **(B, D, F, H)** show outputs based on the parameterization obtained using PyBioNetFit, as described in Materials and Methods. Constraints used in parameterization are represented as colored glyphs above each panel and match the corresponding model variant by color. Vertical black dotted lines indicate the time points at which these constraints apply: 300, 1800, and 3600 seconds. For **(B, D, F, H)**l (PyBioNetFit parameterization), numerical annotations indicate the fraction of accepted MCMC samples satisfying each constraint.

To demonstrate a better approach to model parameterization, we formalized qualitative system properties as Biological Property Specification Language (BPSL) statements (21) (**Supplementary Tables 1**–**5**). Then, following the inference approach of Mitra et al. (19), which is elaborated above, we applied, in a single global optimization, a parallelized metaheuristic optimization method implemented in PyBioNetFit (21) to find maximum likelihood estimates (MLEs) for 28 model parameters and 3 scaling factors that relate model outputs to relative measurements (**Table 3**). The quality of fit to quantitative time-series data (relative measurements) from Kamioka et al. (25) is illustrated in **Figure 1**. The quality of fit to qualitative data—up/down assays relative to a reference—from Catalanotti et al. (24) is illustrated in **Figures 2**, **3**.

In **Figure 1**, the left panel (**Figure 1A**) shows the original fit of Kocieniewski and Lipniacki (23) to time-series data. The right panel (**Figure 1B**) shows the comparable fit found in this study. Our automated parameterization achieves comparable fits across all time courses, with slightly improved agreement for SOS1 and ERK phosphorylation. This improvement is supported quantitatively by the RMSE values reported in **Supplementary Tables 8**, **9**, which show overall lower errors for our MLE parameter estimates relative to the original values from Kocieniewski and Lipniacki (23). Note that the model outputs underlying the relative quantities plotted in **Figure 1** are the absolute cellular abundances of phosphorylated EGFR, SOS1, and ERK. These quantities are each multiplied by an adjustable scaling factor to align with the relative measurements reported by Kamioka et al. (25). Finally, objective function score outputs from PyBioNetFit’s sum of squares (sos) objective function are available in **Supplementary Table 7**. PyBioNetFit’s (**Figure 1B**) final objective function score during optimization is 24.0, where Kocieniewski and Lipniacki’s (23) final objective function score is 40.0.

The curves in **Figures 2**, **3** show calibrated model-predicted phosphorylated MEK and ERK, respectively, as a function of time in different cell lines, as indicated by color and pattern. At the time points corresponding to dotted vertical lines, readouts in pairs of distinct cell lines were compared, resulting in up or down scoring (24). The empirical outcomes of these comparisons are indicated graphically above the dotted vertical lines. As in **Figure 1**, the panels at left (panels A, C, E, and G in both **Figures 2**, **3**) show the simulation results of Kocieniewski and Lipniacki (23), and the panels at right (panels B, D, F, and H in both **Figures 2**, **3**) show the simulation results of this study. As can be seen, we obtained comparable consistency with empirical up/down scoring. Indeed, the calibrated model-predicted time-courses from this study and the earlier study are remarkably similar, despite differences in parameter estimates. To further show that PyBioNetFit’s parameterization of the MEK isoform models maintained comparable consistency to Kocieniewski and Lipniacki’s (23) parameterization of the models, both the original parameterization and PyBioNetFit’s parameterization satisfied 84/90 BPSL constraints determined from distinct cell line readouts from Catalanotti et al. (24).

We note that **Figures 2**, **3** only consider comparisons of readouts in different cell lines. Additional qualitative data were considered in fitting. These data were generated from comparisons of readouts at different time points within the same cellular background. **Supplementary Tables 1**–**5** provide a full listing of the qualitative observations, formalized as BPSL statements, used in fitting.

**Figure 4:**
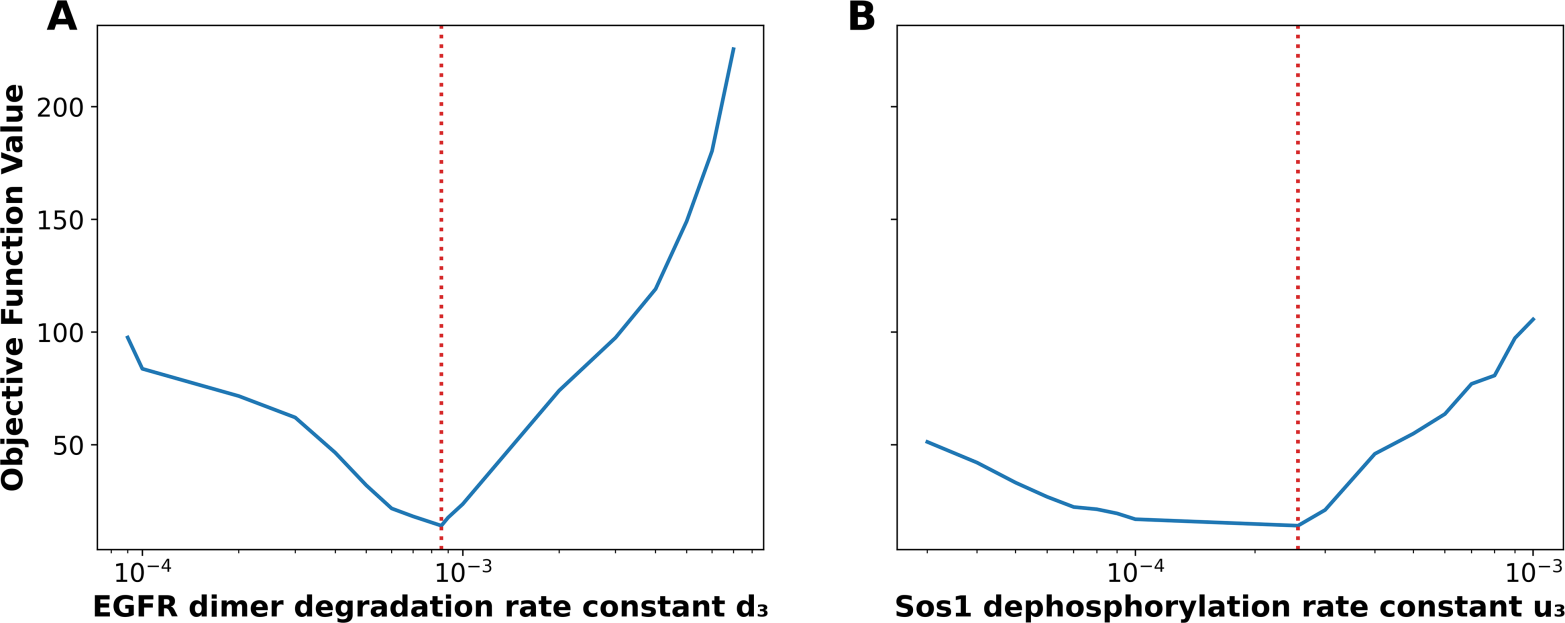
Profile likelihood analysis. **(A)** displays the profile likelihood for the EGFR dimer degradation rate constant $d_{3}$​, and **(B)** displays the profile likelihood for the SOS1 dephosphorylation rate constant $u_{3}$​. Each solid blue curve reports the minimum objective function value found in PyBioNetFit-enabled optimization when the indicated parameter, $d_{3}$ or $u_{3}$, is held fixed at the value indicated on the horizontal axis and the remaining parameters are allowed to vary. The vertical red dashed line in each panel indicates the MLE.

Given the discrepancy between the parameter estimates found here and those of the original study, questions of parametric uncertainty naturally arise. To illustrate that our approach allows for uncertainty quantification (UQ), we generated profile likelihood plots for two rate constants in the models, $d_{3}$ and $u_{3}$ (**Figure 4**). These plots show that both parameters are practically identifiable and that estimation of the value for $d_{3}$ is more constrained by the available data than is estimation of the value for $u_{3}$. In other words, the width of any horizontal line segment interior to the profile of **Figure 4A** (solid blue curve) is shorter than the corresponding line segment in **Figure 4B**. Note that the dotted red vertical line in each panel marks the best-fit estimate.

**Figure 5:**
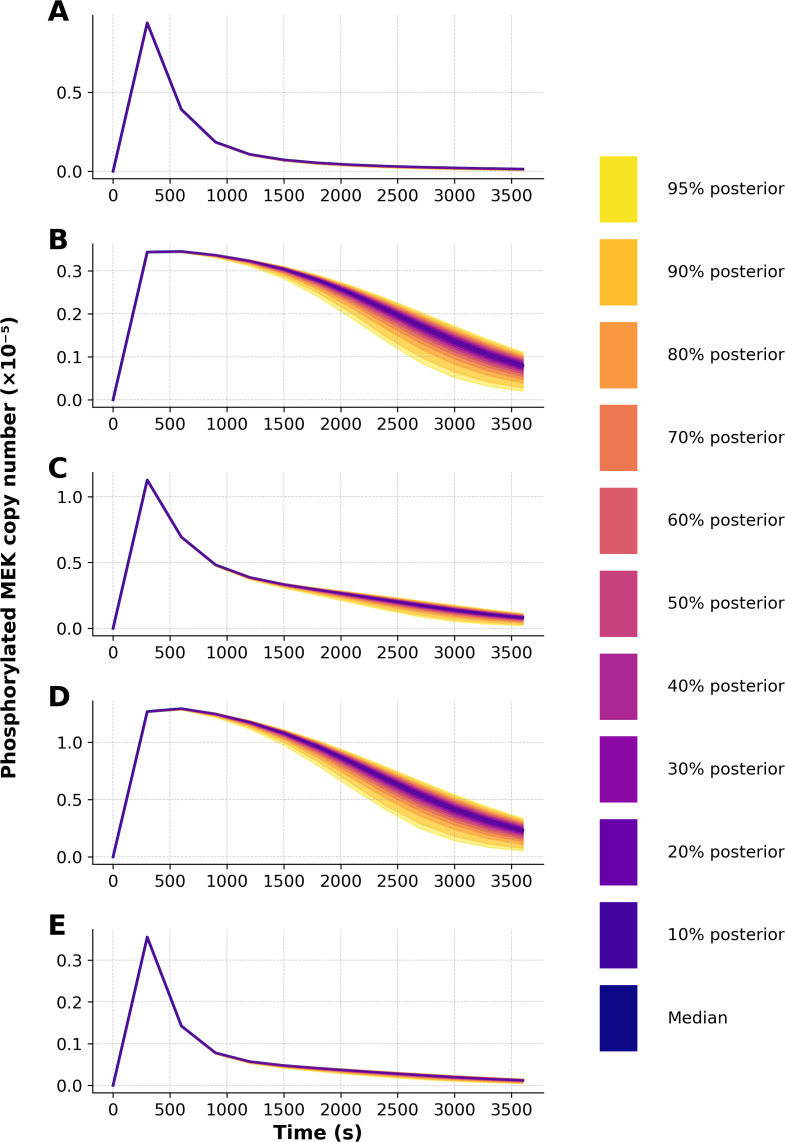
Posterior predictive distributions of phosphorylated MEK copy number for each of the five model variants. Each panel corresponds to one model, as follows: **(A)** WT, **(B)** KO, **(C)** N78G, **(D)** T292A, and **(E)** T292D. The dark blue lines represent medians. Shaded bands indicate credible intervals from 10% to 95%, with the outermost band corresponding to the 95% credible interval. The distribution shown at each time was obtained by propagating parametric uncertainty through simulations, without injection of noise from our noise model.

**Figure 6:**
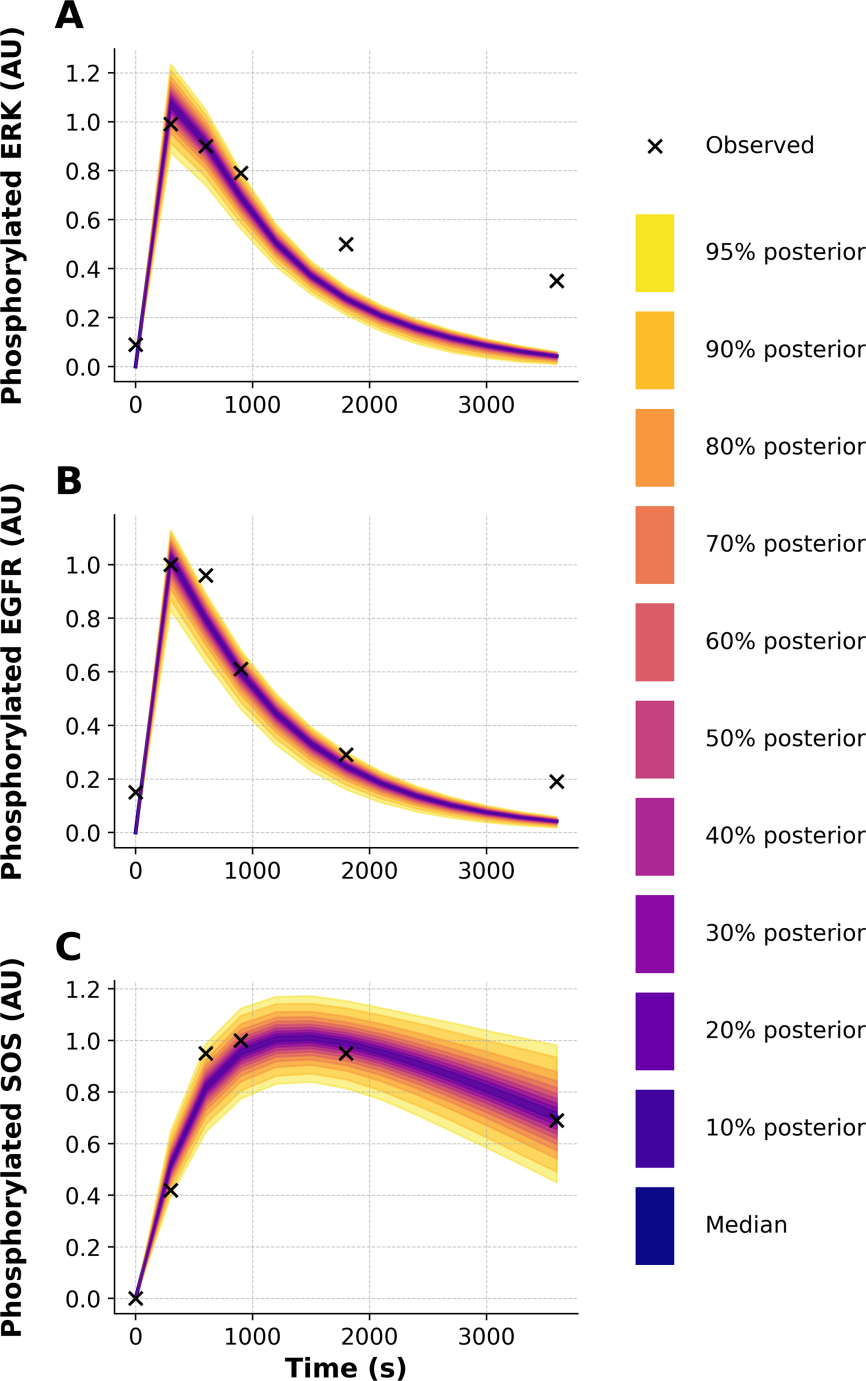
Posterior predictive distributions of phosphorylation outputs of the WT model: **(A)** phosphorylated ERK, **(B)** phosphorylated EGFR, and **(C)** phosphorylated SOS1, all expressed in arbitrary units (AU). The distribution shown at each time point was obtained by propagating parametric uncertainty through simulations, without injection of noise from our noise model. Experimental data are included in each panel. Dark blue lines represent median trajectories, and colored bands indicate credible intervals from 10% to 95%, with the outermost band corresponding to the 95% credible interval.

**Figure 7:**
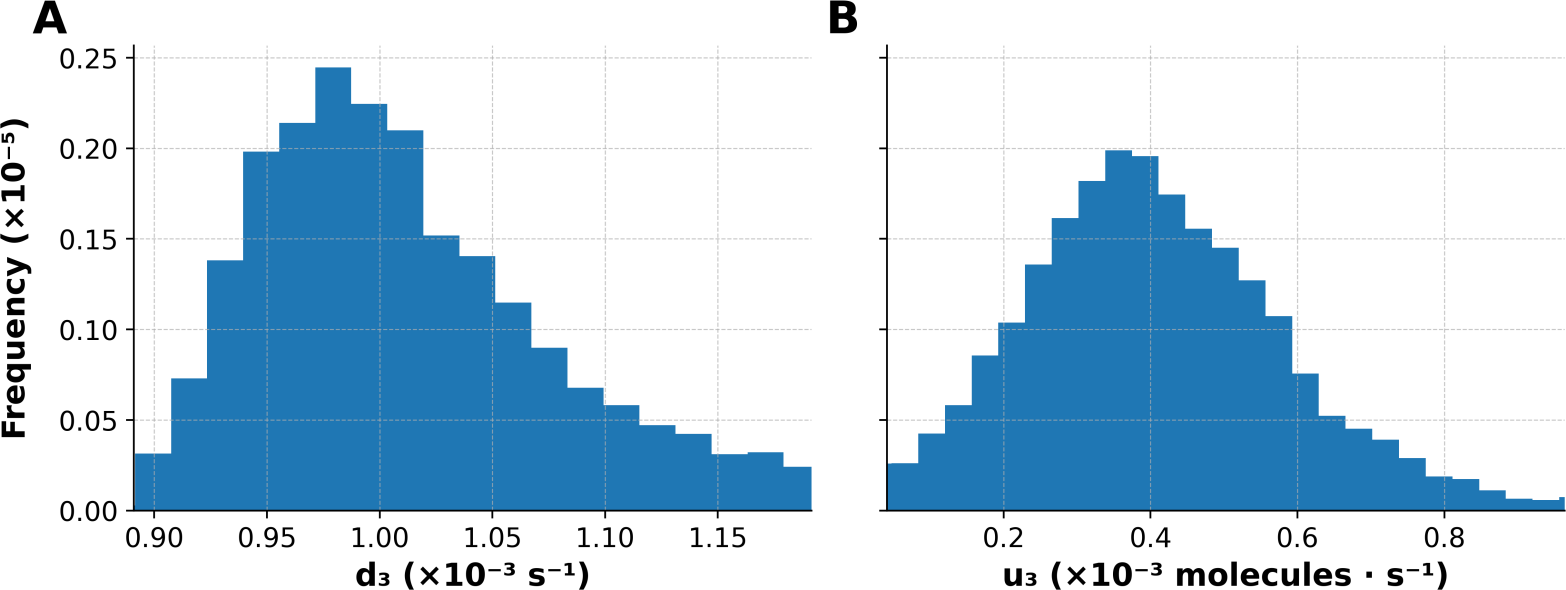
Marginal posterior distributions for the rate constants **(A)** $d_{3}$ and **(B)** $u_{3}$. These histograms were derived from samples generated using the adaptive MCMC sampler implemented in PyBioNetFit, as described in Materials and Methods. Parametric uncertainty is characterized by histogram width.

To further demonstrate the capabilities of PyBioNetFit, we executed a Bayesian approach to inference and UQ, which leveraged the same datasets as those considered in maximum likelihood estimation. However, for the sake of simplicity, we focused on a smaller set of adjustable parameters. Through Markov chain Monte Carlo (MCMC) sampling, we were able to obtain samples converging on and representing the parameter posterior distribution. Sampling convergence metrics are reported in **Table 4**. Additional convergence diagnostics, likelihood and parameter trace plots and pairs plots, are shown in **Supplementary Figures 1**–**3**. Marginal posterior distributions for the rate constants $d_{3}$ and $u_{3}$ are shown in **Figure 5**. By propagating parametric uncertainty through simulations, we obtained posterior predictive distributions characterizing uncertainty in model predictions (**Figures 6**, **7**). **Supplementary Table 6** quantifies consistency with qualitative observations.

**Table 4:** Sampling convergence metrics​.

Parameter	${\text{ESS}}_{\text{Bulk}}$	${\text{ESS}}_{\text{Tail}}$	$\hat{R}-1$
$d_{3}$	6160	8620	$1.8x10^{-4}$
$u_{3}$	2150	2120	$6.0x10^{-4}$

For the rate constants $d_{3}$ and $u_{3}$, the table reports the *bulk* and *tail* Effective Sample Size (ESS), as well as the quantity $\hat{R}-1$, in which $\hat{R}$ is a convergence diagnostic derived from the potential scale reduction factor and computed using the rstan R package. ${\text{ESS}}_{\text{Bulk}}$ evaluates the effective number of independent samples for the central bulk of the posterior distribution, whereas ${\text{ESS}}_{\text{Tail}}$ reflects the stability of sampling in the distribution tails. High ESS values across both metrics indicate efficient sampling with low autocorrelation. The $\hat{R}-1$ values are close to zero, suggesting convergence of the chains and well-mixed posterior estimates.

## 4 Discussion

In this study, we demonstrated that PyBioNetFit (21) can replace manual trial-and-error parameter tuning, as in the study of Kocieniewski and Lipniacki (23), with a reproducible, automated pipeline that integrates both quantitative and qualitative data. We showed how to formalize up/down results of case-control comparisons as Biological Property Specification Language (BPSL) statements, and we showed how to leverage BPSL statements in concert with conventional time-series data in not only model parameterization but also rigorous uncertainty quantification (UQ). We elaborated on the approach of Mitra et al. (19), showing that this approach is grounded in likelihood-based inference. We also demonstrated both frequentist and Bayesian approaches to rigorous uncertainty quantification (UQ), which was missing in the original modeling study of Kocieniewski and Lipniacki (23).

An important aspect of our re-parameterization of Kocieniewski and Lipniacki’s (23) MEK isoform models is that different parameter sets can yield comparably good fits to the available data. This phenomenon arises when multiple combinations of parameters produce near-optimal objective function values. Lack of organization in models like these can be closely related to practical non-identifiability, in which parameter estimates cannot be uniquely determined given the data. In our profile likelihood analysis of all 28 fitted parameters, we found that approximately half were practically identifiable, while the remainder showed broad, flat profiles, indicating limited sensitivity to perturbation. The full results are shown in **Supplementary Figure 4**, which plots each parameter against its objective function profile, maximum likelihood estimate, and deviation from the original baseline from Kocieniewski and Lipniacki (23). Although this lack of uniqueness highlights the need for additional experimental data to fully constrain the system, it is important to note that model predictions remained consistent across different parameter sets. Thus, despite non-identifiable parameters, the model exhibits robustness, supporting the reliability of the conclusions drawn from our PyBioNetFit-enabled analysis.

Our results illustrate several advantages of a PyBioNetFit-enabled workflow, including potential for straightforward reusability of qualitative observations, once formalized as BPSL statements, and reproducibility of complex workflows. PyBioNetFit job setup files consist of easily shared plain-text files capturing data, models, and algorithms in standardized formats. Our results also reinforce the value of qualitative data. These data can be used in model parameterization with UQ, in multiple ways. Looking ahead, this study serves as a template for enhancing model parameterization pipelines in contexts where qualitative data are available to complement (limited) quantitative data.

### 4.1 Limitations and future directions

While our study demonstrates the utility of PyBioNetFit for automated parameterization with both qualitative and quantitative data, several limitations should be acknowledged. First, scalability remains a practical challenge. Although PyBioNetFit performs well for models of moderate size, applying it to models with hundreds or thousands of parameters, especially those that are high-dimensional, will become too computationally expensive. Future work may benefit from hybrid optimization strategies that combine global search with local refinement to improve efficiency.

Secondly, while the Biological Property Specification Language (BPSL) is well suited to encoding rank orderings, inequalities at fixed times, and cross-condition comparisons, it does not currently support constraints on more complex temporal features, such as peak times. Extending BPSL to represent such properties, as well as improving its ability to incorporate metadata (e.g., experimental conditions, cell types), would expand its applicability.

Third, BPSL has potential beyond parameter estimation. For example, BPSL statements could be leveraged in model-checking workflows to systematically document a model’s consistency with qualitative biological knowledge. In addition, advances in natural language processing, particularly large language models, could enable automated extraction of BPSL statements from full-text articles, further broadening their utility in parameterization and uncertainty quantification workflows.

Taken together, these considerations highlight important directions for future research. Our demonstration emphasizes that while current approaches are powerful, there is significant scope for enhancing both scalability and expressivity, thereby enabling broader adoption of hybrid data workflows in systems biology.

## Data Availability

The original contributions presented in the study are included in the article/**Supplementary Material**. Further inquiries can be directed to the corresponding authors.

## References

[1] Mitra ED Hlavacek WS . Parameter estimation and uncertainty quantification for systems biology models. Curr Opin Syst Biol. (2019) 18:9–18. doi: 10.1016/j.coisb.2019.10.006. PMID: 32719822 PMC7384601

[2] Chen KC Csikasz-Nagy A Gyorffy B Val J Novak B Tyson JJ . Kinetic analysis of a molecular model of the budding yeast cell cycle. Mol Biol Cell. (2000) 11:369–91. doi: 10.1091/mbc.11.1.369. PMID: 10637314 PMC14780

[3] Chen KC Calzone L Csikasz-Nagy A Cross FR Novak B Tyson JJ . Integrative analysis of cell cycle control in budding yeast. Mol Biol Cell. (2004) 15:3841–62. doi: 10.1091/mbc.e03-11-0794. PMID: 15169868 PMC491841

[4] Kraikivski P Chen KC Laomettachit T Murali TM Tyson JJ . From START to FINISH: computational analysis of cell cycle control in budding yeast. NPJ Syst Biol Appl. (2015) 1:1–9. doi: 10.1038/npjsba.2015.16. PMID: 28725464 PMC5516803

[5] Barik D Ball DA Peccoud J Tyson JJ . A stochastic model of the yeast cell cycle reveals roles for feedback regulation in limiting cellular variability. PloS Comput Biol. (2016) 12:e1005230. doi: 10.1371/journal.pcbi.1005230. PMID: 27935947 PMC5147779

[6] Tyson JJ Laomettachit T Kraikivski P . Modeling the dynamic behavior of biochemical regulatory networks. J Theor Biol. (2019) 462:514–27. doi: 10.1016/j.jtbi.2018.11.034. PMID: 30502409 PMC6369921

[7] Kinney JB Tkacik G Callan CG . Precise physical models of protein-DNA interaction from high-throughput data. Proc Natl Acad Sci USA. (2007) 104:501–6. doi: 10.1073/pnas.0609908104. PMID: 17197415 PMC1766414

[8] Kinney JB Murugan A Gallan CG Cox EC . Using deep sequencing to characterize the biophysical mechanism of a transcriptional regulatory sequence. Proc Natl Acad Sci USA. (2010) 107:9158–63. doi: 10.1073/pnas.1004290107. PMID: 20439748 PMC2889059

[9] Atwal GS Kinney JB . Learning quantitative sequence–function relationships from massively parallel experiments. J Stat Phys. (2016) 162:1203–43. doi: 10.1007/s10955-015-1398-3. PMID: 41933263

[10] Kinney JB McCandlish DM . Massively parallel assays and quantitative sequence-function relationships. Annu Rev Genomics Hum Genet. (2019) 20:99–127. doi: 10.1146/annurev-genom-083118-014845. PMID: 31091417

[11] Toni T Jovanovic G Huvet M Buck M Stumpf MPH . From qualitative data to quantitative models: analysis of the phage shock protein stress response in Escherichia coli. BMC Syst Biol. (2011) 5:69. doi: 10.1186/1752-0509-5-69. PMID: 21569396 PMC3127791

[12] Pargett M Umulis DM . Quantitative model analysis with diverse biological data: applications in developmental pattern formation. Methods. (2013) 62:56–67. doi: 10.1016/j.ymeth.2013.03.024. PMID: 23557990

[13] Pargett M Rundell AE Buzzard GT Umulis DM . Model-based analysis for qualitative data: an application in Drosophila germline stem cell regulation. PloS Comput Biol. (2014) 10:e1003498. doi: 10.1371/journal.pcbi.1003498. PMID: 24626201 PMC3952817

[14] Schmiester L Weindl D Hasenauer J . Parameterization of mechanistic models from qualitative data using an efficient optimal scaling approach. J Math. Biol. (2020) 81:603–23. doi: 10.1007/s00285-020-01522-w. PMID: 32696085 PMC7427713

[15] Schmiester L Weindl D Hasenauer J . Efficient gradient-based parameter estimation for dynamic models using qualitative data. Bioinformatics. (2021) 37:4493–500. doi: 10.1093/bioinformatics/btab512. PMID: 34260697 PMC8652033

[16] Dorešić D Grein S Hasenauer J . Efficient parameter estimation for ODE models of cellular processes using semi-quantitative data. Bioinformatics. (2024) 40:i558–66:btae210. doi: 10.1093/bioinformatics/btae210, PMID: 38940161 PMC11211815

[17] Tareen A Kooshkbaghi M Posfai A Ireland WT McCandlish DM Kinney JB . MAVE-NN: learning genotype-phenotype maps from multiplex assays of variant effect. Genome Biol. (2022) 23:98. doi: 10.1186/s13059-022-02661-7, PMID: 35428271 PMC9011994

[18] Oguz C Laomettachit T Chen KC Watson LT Baumann WT Tyson JJ . Optimization and model reduction in the high dimensional parameter space of a budding yeast cell cycle model. BMC Syst Biol. (2013) 7:1–17. doi: 10.1186/1752-0509-7-53. PMID: 23809412 PMC3702416

[19] Mitra ED Dias R Posner RG Hlavacek WS . Using both qualitative and quantitative data in parameter identification for systems biology models. Nat Commun. (2018) 9:3901. doi: 10.1038/s41467-018-06439-z. PMID: 30254246 PMC6156341

[20] Mitra ED Hlavacek WS . Bayesian inference using qualitative observations of underlying continuous variables. Bioinformatics. (2020) 36:3177–84. doi: 10.1093/bioinformatics/btaa084. PMID: 32049328 PMC7214020

[21] Mitra ED Suderman R Colvin J Ionkov A Hu A Sauro HM . PyBioNetFit and the Biological Property Specification Language. iScience. (2019) 19:1012–36. doi: 10.2139/ssrn.3382545. PMID: 31522114 PMC6744527

[22] Schälte Y Fröhlich F Jost PJ Vanhoefer J Pathirana D Stapor P . pyPESTO: A modular and scalable tool for parameter estimation for dynamic models. Bioinformatics. (2023) 39:btad711. doi: 10.1093/bioinformatics/btad711, PMID: 37995297 PMC10689677

[23] Kocieniewski P Lipniacki T . MEK1 and MEK2 differentially control the duration and amplitude of the ERK cascade response. Phys Biol. (2013) 10:35006. doi: 10.1088/1478-3975/10/3/035006. PMID: 23735655

[24] Catalanotti F Reyes G Jesenberger V Galabova-Kovacs G de Matos Simoes R Carugo O . A Mek1–Mek2 heterodimer determines the strength and duration of the Erk signal. Nat Struct Mol Biol. (2009) 16:294–303. doi: 10.1038/nsmb.1564. PMID: 19219045

[25] Kamioka Y Yasuda S Fujita Y Aoki K Matsuda M . Multiple decisive phosphorylation sites for the negative feedback regulation of SOS1 via ERK. J Biol Chem. (2010) 285:33540–8. doi: 10.1074/jbc.m110.135517. PMID: 20724475 PMC2963383

[26] Kreutz C Raue A Kaschek D Timmer J . Profile likelihood in systems biology. FEBS J. (2013) 280:2564–71. doi: 10.1111/febs.12276. PMID: 23581573

[27] Andrieu C Thoms J . A tutorial on adaptive MCMC. Stat Comp. (2008) 18:343–73. doi: 10.1007/s11222-008-9110-y. PMID: 41933263

[28] Neumann J Lin YT Mallela A Miller EF Colvin J Duprat AT . Implementation of a practical Markov chain Monte Carlo sampling algorithm in PyBioNetFit. Bioinformatics. (2022) 38:1770–2. doi: 10.1093/bioinformatics/btac004. PMID: 34986226 PMC10060707

[29] Faeder JR Blinov ML Hlavacek WS . Rule-based modeling of biochemical systems with BioNetGen. Methods Mol Biol. (2009) 500:113–67. doi: 10.1007/978-1-59745-525-1_5. PMID: 19399430

[30] Keating SM Waltemath D König M Zhang F Dräger A Chaouiya C . SBML Level 3: an extensible format for the exchange and reuse of biological models. Mol Syst Biol. (2020) 16:e9110. doi: 10.15252/msb.20199110. PMID: 32845085 PMC8411907

[31] Harris LA Hogg JS Tapia JJ Sekar JAP Gupta S Korsunsky I . BioNetGen 2.2: advances in rule-based modeling. Bioinformatics. (2016) 32:3366–8. doi: 10.1093/bioinformatics/btw469. PMID: 27402907 PMC5079481

[32] Vehtari A Gelman A Simpson D Carpenter B Bürkner PC . Rank-normalization, folding, and localization: an improved <math><mover accent=‘true’><mi>R</mi><mo>^</mo></mover></math> for assessing convergence of MCMC (with discussion). Bayesian. Anal. (2021) 16:667–718. doi: 10.1214/20-ba1221. PMID: 41486833

