# Supplementary material for: Using PyBioNetFit to leverage qualitative and quantitative data in biological model parameterization and uncertainty quantification
Source: Front Immunol. 2026 Apr 29;17:1663008. doi: 10.3389/fimmu.2026.1663008 (PMC13168053; doi:10.3389/fimmu.2026.1663008)
Supplement: Supplementary file 1 [file DataSheet1.pdf]

Using PyBioNetFit to Leverage Qualitative and Quantitative Data in Biological Model Parameterization and Uncertainty Quantification

Ely F. Miller[1†], Abhishek Mallela[2,3†], Jacob Neumann[4], Yen Ting Lin[2,5], William S. Hlavacek[2,3*], Richard G. Posner[1*]

[1]Department of Biological Sciences, Northern Arizona University, Flagstaff, Arizona, United States of America

[2]Center for Nonlinear Studies, Los Alamos National Laboratory, Los Alamos, New Mexico, United States of America

[3]Theoretical Biology and Biophysics Group, Theoretical Division, Los Alamos National Laboratory, Los Alamos, New Mexico, United States of America

[4]Department of Chemistry and Chemical Biology, Cornell University, Ithaca, New York, United States of America

[5]Information Sciences Group, Computer, Computational and Statistical Sciences Division, Los Alamos National Laboratory, Los Alamos, New Mexico, United States of America

[†] These authors contributed equally.

* Address correspondence to W.S.H. (hlavacek@lanl.gov) or R.G.P. (richard.posner@nau.edu).

1 Glossary of Terms and Abbreviations

BPSL (Biological Property Specification Language): A formal language used to encode qualitative biological constraints (e.g., comparisons of time-course values between species or conditions).

Constraint (C#): A numbered qualitative rule (C1, C2, etc.) expressed in BPSL, used to test whether model simulations match expected biological behaviors. Constraint glossaries for each model are provided in Supplemental Tables 1–5.

DE (Differential Evolution): A global optimization algorithm used in PyBioNetFit for parameter estimation.

Isoform Models (WT, KO, N78G, T292A, T292D): Variants of the MEK signaling pathway model.

- **WT:** Wild-type model.
- **KO:** Knockout model (gene disrupted).
- **N78G:** MEK isoform model with the N78G mutation.
- **T292A:** MEK isoform model with the T292A mutation.
- **T292D:** MEK isoform model with the T292D mutation.

MLE (Maximum Likelihood Estimation): Statistical method for estimating parameters by maximizing the likelihood of the observed data given the model.

MCMC (Markov Chain Monte Carlo): Sampling method used to explore posterior distributions in Bayesian inference.

Objective Function (SOS): In PyBioNetFit, the Sum of Squares (SOS) objective function measures the difference between model predictions and data; lower scores indicate better fits.

pERK1/2: Phosphorylated ERK1/2, a key downstream signaling protein measured in the models.

pMEK (MEK_pRDS): Phosphorylated MEK (measured as relative densitometry signal, RDS) in experimental datasets and model outputs.

pSOS1, pEGFR, pERK: Phosphorylated forms of signaling proteins SOS1, EGFR, and ERK, respectively; used for quantitative comparison between experiment and simulation.

Profile Likelihood Analysis: A method to assess parameter identifiability by varying one parameter at a time while re-optimizing others, producing likelihood profiles.

PyBioNetFit: Software framework used for parameterization and uncertainty quantification of biological models using quantitative data and qualitative constraints.

RMSE (Root Mean Squared Error): A measure of model fit, quantifying the average difference between model outputs and experimental data; lower values indicate better fits.

2 Supplementary Data

The source code used in this study can be found in the PyBioNetFit GitHub repository (https://github.com/lanl/PyBNF). Within this repository, the datasets and job setup files used in this study for maximum likelihood estimation and Bayesian inference can be found here: https://github.com/lanl/PyBNF/tree/master/examples/Miller2025_MEK_Isoforms

3 Supplementary Tables and Figures

Constraint #	WT Model
C1	WT.MEK_pRDS at time=300 > WT.MEK_pRDS at time=1800
C2	WT.MEK_pRDS at time=1800 > WT.MEK_pRDS at time=3600
C3	WT.MEK_pRDS at time=300 > WT.MEK_pRDS at time=3600
C4	WT.MEK_pRDS at time=300 > KO.MEK_pRDS at time=300
C5	WT.MEK_pRDS at time=1800 < KO.MEK_pRDS at time=1800
C6	WT.MEK_pRDS at time=3600 < KO.MEK_pRDS at time=3600
C7	WT.MEK_pRDS at time=300 < N78G.MEK_pRDS at time=300
C8	WT.MEK_pRDS at time=1800 < N78G.MEK_pRDS at time=1800
C9	WT.MEK_pRDS at time=3600 < N78G.MEK_pRDS at time=3600
C10	WT.MEK_pRDS at time=300 < T292A.MEK_pRDS at time=300

C11	WT.MEK_pRDS at time=1800 < T292A.MEK_pRDS at time=1800
C12	WT.MEK_pRDS at time=3600 < T292A.MEK_pRDS at time=3600
C13	WT.MEK_pRDS at time=300 > T292D.MEK_pRDS at time=300
C14	WT.MEK_pRDS at time=1800 > T292D.MEK_pRDS at time=1800
C15	WT.MEK_pRDS at time=3600 > T292D.MEK_pRDS at time=3600
C16	WT.pERK1_2 at time=300 > WT.pERK1_2 at time=1800
C17	WT.pERK1_2 at time=1800 > WT.pERK1_2 at time=3600
C18	WT.pERK1_2_ at time=300 > WT.pERK1_2 at time=3600
C19	WT.pERK1_2 at time=300 > KO.pERK1_2 at time=300
C20	WT.pERK1_2 at time=1800 < KO.pERK1_2 at time=1800
C21	WT.pERK1_2 at time=3600 < KO.pERK1_2 at time=3600
C22	WT.pERK1_2 at time=300 > N78G.pERK1_2 at time=300
C23	WT.pERK1_2 at time=1800 < N78G.pERK1_2 at time=1800
C24	WT.pERK1_2 at time=3600 < N78G.pERK1_2 at time=3600
C25	WT.pERK1_2 at time=300 < T292A.pERK1_2 at time=300
C26	WT.pERK1_2 at time=1800 < T292A.pERK1_2 at time=1800
C27	WT.pERK1_2 at time=3600 < T292A.pERK1_2 at time=3600
C28	WT.pERK1_2 at time=300 > T292D.pERK1_2 at time=300
C29	WT.pERK1_2 at time=1800 > T292D.pERK1_2 at time=1800
C30	WT.pERK1_2 at time=3600 > T292D.pERK1_2 at time=3600

Supplemental Table 1 (WT constraint glossary):
This table provides a glossary for the constraint labels (C1–C30) used in Supplemental Table 6 for the WT model. Each entry is a BPSL statement that formalizes a qualitative observation. Time points in the statements are given in seconds.

Constraint #	KO Model
C1	KO.MEK_pRDS at time=300 > KO.MEK_pRDS at time=1800
C2	KO.MEK_pRDS at time=300 > KO.MEK_pRDS at time=3600
C3	KO.MEK_pRDS at time=1800 > KO.MEK_pRDS at time=3600
C4	KO.MEK_pRDS at time=300 < N78G.MEK_pRDS at time=300
C5	KO.MEK_pRDS at time=1800 < N78G.MEK_pRDS at time=1800
C6	KO.MEK_pRDS at time=3600 > N78G.MEK_pRDS at time=3600
C7	KO.MEK_pRDS at time=300 < T292A.MEK_pRDS at time=300
C8	KO.MEK_pRDS at time=1800 < T292A.MEK_pRDS at time=1800
C9	KO.MEK_pRDS at time=3600 < T292A.MEK_pRDS at time=3600
C10	KO.MEK_pRDS at time=300 > T292D.MEK_pRDS at time=300
C11	KO.MEK_pRDS at time=1800 > T292D.MEK_pRDS at time=1800

C12	KO.MEK_pRDS at time=3600 > T292D.MEK_pRDS at time=3600
C13	KO.pERK1_2 at time=300 > KO.pERK1_2at time=1800
C14	KO.pERK1_2 at time=1800 > KO.pERK1_2 at time=3600
C15	KO.pERK1_2 at time=300 > KO.pERK1_2 at time=3600
C16	KO.pERK1_2 at time=300 < N78G.pERK1_2 at time=300
C17	KO.pERK1_2 at time=1800 < N78G.pERK1_2 at time=1800
C18	KO.pERK1_2 at time=3600 < N78G.pERK1_2 at time=3600
C19	KO.pERK1_2 at time=300 < T292A.pERK1_2 at time=300
C20	KO.pERK1_2 at time=1800 < T292A.pERK1_2 at time=1800
C21	KO.pERK1_2 at time=3600 < T292A.pERK1_2 at time=3600
C22	KO.pERK1_2 at time=300 > T292D.pERK1_2 at time=300
C23	KO.pERK1_2 at time=1800 > T292D.pERK1_2 at time=1800
C24	KO.pERK1_2 at time=3600 > T292D.pERK1_2 at time=3600

Supplemental Table 2 (KO constraint glossary):
This table provides a glossary for the constraint labels (C1–C24) used in Supplemental Table 6 for the KO model. Each entry is a BPSL statement that formalizes a qualitative observation. Time points in the statements are given in seconds.

\

Constraint #	N78G Model
C1	N78G.MEK_pRDS at time=300 > N78G.MEK_pRDS at time=1800
C2	N78G.MEK_pRDS at time=1800 > N78G.MEK_pRDS at time=3600
C3	N78G.MEK_pRDS at time=300 > N78G.MEK_pRDS at time=3600
C4	N78G.MEK_pRDS at time=300 < T292A.MEK_pRDS at time=300
C5	N78G.MEK_pRDS at time=1800 < T292A.MEK_pRDS at time=1800
C6	N78G.MEK_pRDS at time=3600 < T292A.MEK_pRDS at time=3600
C7	N78G.MEK_pRDS at time=300 > T292D.MEK_pRDS at time=300
C8	N78G.MEK_pRDS at time=1800 > T292D.MEK_pRDS at time=1800
C9	N78G.MEK_pRDS at time=3600 > T292D.MEK_pRDS at time=3600
C10	N78G.pERK1_2 at time=300 > N78G.pERK1_2 at time=1800
C11	N78G.pERK1_2 at time=1800 > N78G.pERK1_2 at time=3600
C12	N78G.pERK1_2 at time=300 > N78G.pERK1_2 at time=3600

C13	N78G.pERK1_2 at time=300 < T292A.pERK1_2 at time=300
C14	N78G.pERK1_2 at time=1800 < T292A.pERK1_2 at time=1800
C15	N78G.pERK1_2 at time=3600 < T292A.pERK1_2 at time=3600
C16	N78G.pERK1_2 at time=300 > T292D.pERK1_2 at time=300
C17	N78G.pERK1_2 at time=1800 > T292D.pERK1_2 at time=1800
C18	N78G.pERK1_2 at time=3600 > T292D.pERK1_2 at time=3600

Supplemental Table 3 (N78G constraint glossary):
This table provides a glossary for the constraint labels (C1–C18) used in Supplemental Table 6 for the N78G model. Each entry is a BPSL statement that formalizes a qualitative observation. Time points in the statements are given in seconds.

Constraint #	T292A Model
C1	T292A.MEK_pRDS at time=300 > T292A.MEK_pRDS at time=1800
C2	T292A.MEK_pRDS at time=300 > T292A.MEK_pRDS at time=3600
C3	T292A.MEK_pRDS at time=1800 > T292A.MEK_pRDS at time=3600
C4	T292A.MEK_pRDS at time=300 > T292D.MEK_pRDS at time=300
C5	T292A.MEK_pRDS at time=1800 > T292D.MEK_pRDS at time=1800
C6	T292A.MEK_pRDS at time=3600 > T292D.MEK_pRDS at time=3600
C7	T292A.pERK1_2 at time=300 > T292A.pERK1_2 at time=1800
C8	T292A.pERK1_2 at time=1800 > T292A.pERK1_2 at time=3600
C9	T292A.pERK1_2 at time=300 > T292A.pERK1_2 at time=3600
C10	T292A.pERK1_2 at time=300 > T292D.pERK1_2 at time=300
C11	T292A.pERK1_2 at time=1800 > T292D.pERK1_2 at time=1800
C12	T292A.pERK1_2 at time=3600 > T292D.pERK1_2 at time=3600

Supplemental Table 4 (T292A constraint glossary):
This table provides a glossary for the constraint labels (C1–C12) used in Supplemental Table 6 for

the T292A model. Each entry is a BPSL statement that formalizes a qualitative observation. Time points in the statements are given in seconds.

Constraint #	T292D Model
C1	T292D.MEK_pRDS at time=300 > T292D.MEK_pRDS at time=1800
C2	T292D.MEK_pRDS at time=300 > T292D.MEK_pRDS at time=3600
C3	T292D.MEK_pRDS at time=1800 > T292D.MEK_pRDS at time=3600
C4	T292D.pERK1_2 at time=300 > T292D.pERK1_2 at time=1800
C5	T292D.pERK1_2 at time=1800 > T292D.pERK1_2 at time=3600
C6	T292D.pERK1_2 at time=300 > T292D.pERK1_2 at time=3600

Supplemental Table 5 (T929D constraint glossary):
This table provides a glossary for the constraint labels (C1–C30) used in Supplemental Table 6 for the T292D model. Each entry is a BPSL statement that formalizes a qualitative observation. Time points in the statements are given in seconds.

Constraint #	WT Model	KO Model	N78G Model	T292A Model	T292D Model
C1	100	100	100	100	100
C2	100	100	100	100	100
C3	100	100	100	100	100
C4	100	100	100	100	100
C5	100	100	100	100	0
C6	100	0.2	100	100	100
C7	100	100	100	100	--
C8	100	100	100	100	--
C9	100	100	100	100	--
C10	100	0	100	100	--
C11	100	100	100	100	--
C12	100	100	100	100	--
C13	100	100	100	--	--
C14	100	100	100	--	--
C15	99.8	100	100	--	--

C16	100	0	100	--	--
C17	100	0	100	--	--
C18	100	0	100	--	--
C19	0	0	--	--	--
C20	100	0	--	--	--
C21	100	0	--	--	--
C22	0	100	--	--	--
C23	100	100	--	--	--
C24	100	100	--	--	--
C25	100	--	--	--	--
C26	100	--	--	--	--
C27	100	--	--	--	--
C28	100	--	--	--	--
C29	100	--	--	--	--
C30	100	--	--	--	--

Supplemental Table 6: Percentage of accepted MCMC samples that satisfy the indicated constraint. A total of 90 constraints on predicted system behavior were defined using BPSL. Each entry is the percent of sampled parameter values yielding consistency with the indicated constraint. An entry of 100% indicates that a constraint was satisfied for sampled parameter sets, whereas an entry of 0% indicates that the constraint was never satisfied. Constraints are labeled C1–C30 and listed in the same top-down order as in each model's corresponding PROP file. Columns indicate individual models (e.g., the WT model), and rows indicate specific constraints. An entry of "--" indicates that a constraint was not applicable. See Supplemental Tables 1–5 for the BPSL statements that define the constraints.

	Original Parameterization	PyBioNetFit Parameterization
Objective Funtion Score	40.0	24.0

Supplemental Table 7: Overall objective function score for both Kocieniewski and Lipniacki's (2013) parameterization and PyBioNetFit's parameterization. Objective function scores were calculated using PyBioNetFit's Sum of Squares objective function and Differential Evolution fitting algorithm. Lower scores indicate better fits.

**Time (s)**	**Species**	**Experimental Data (AU)**	**RMSE (PyBioNetFit)**	**RMSE (Original)**
**0**	pSOS1	0.0	0.0	0.0
**0**	pEGFR	1.5	1.5	1.5
**0**	pERK	0.9	0.9	0.9
**300**	pSOS1	4.2	1.776	1.846
**300**	pEGFR	10.0	0.105	0.213
**300**	pERK	9.9	0.496	0.028
**600**	pSOS1	9.5	0.539	0.39
**600**	pEGFR	9.6	0.277	1.879
**600**	pERK	9.0	0.245	0.624
**900**	pSOS1	10.0	0.536	0.038
**900**	pEGFR	6.1	0.61	0.258
**900**	pERK	7.9	0.979	1.546

**1800**	pSOS1	9.5	0.251	0.977
**1800**	pEGFR	2.9	0.836	0.429
**1800**	pERK	5.0	0.391	2.368
**3600**	pSOS1	6.9	0.145	2.809
**3600**	pEGFR	1.9	1.579	1.421
**3600**	pERK	3.5	2.984	3.029

Supplemental Table 8: RMSE values for PyBioNetFit outputs vs original parameterization outputs at each time point for the data in the WT.exp file, available in the supplemental setup files in the LANL GitHub. Experimental AU data are compared to model outputs for each species (pSOS1, pEGFR, pERK), at six experimental sampling times, across both PyBioNetFit's parameterization and the original parameterization. The statistic shown is Root Mean Standard Error, highlighting the distance each model's parameterization output from the experimental data points. Lower RMSE scores is associated with a better fit to the data.

Parameterization	Global RMSE
**PyBioNetFit**	1.076
**Original**	1.47

Supplemental Table 9: Global RMSE scores for each model parameterization's output, considering all time points and experimental data points across all 3 species (Sos1, EGFR, ERK) as seen in Supplemental Table 8. Lower RMSE scores indicate a better fit to the experimental data.

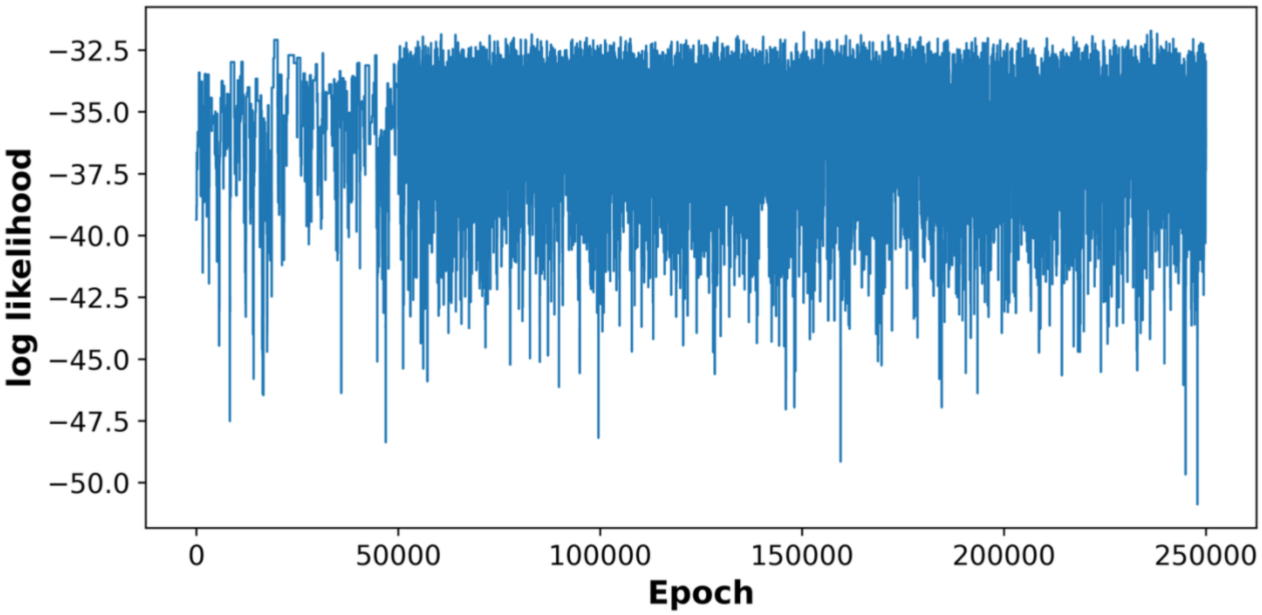

Supplementary Figure 1: Trace plot of log-likelihood values across 250,000 production iterations of MCMC sampling.

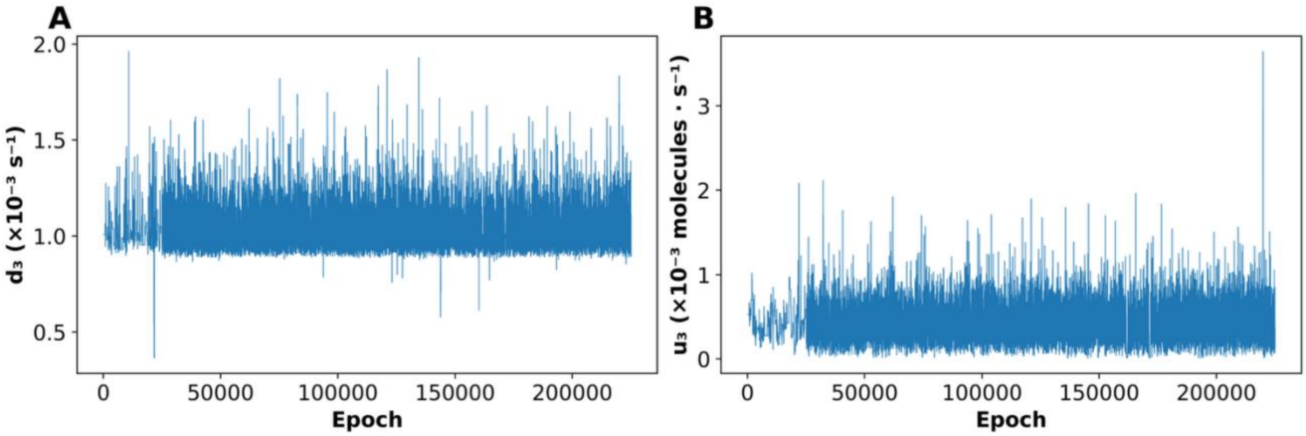

Supplementary Figure 2: Trace plots for each of two parameters across 250,000 iterations of production sampling. Panel A shows the trace plot for d_3, and panel B shows the trace plot for u_3.

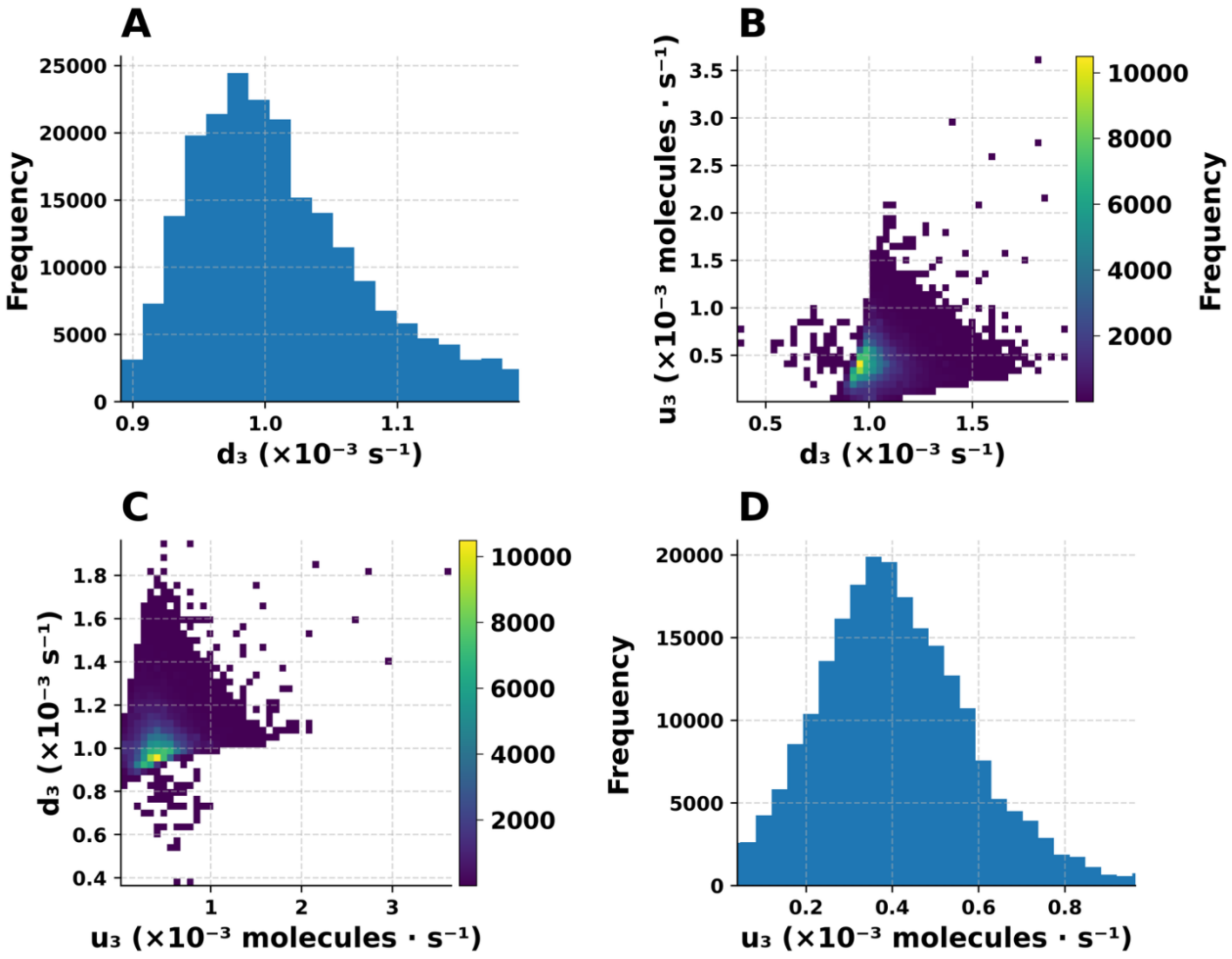

Supplementary Figure 3: Pairs plots of posterior samples for the rate constants d_3 and u_3. (A) Marginal posterior for d_3. (D) Marginal posterior for u_3. (B, C) Joint posterior density, i.e., the continuous two-dimensional density estimate of the posterior over (d_3, u_3). Colors indicate relative sample density, from white (lowest) to yellow (highest).

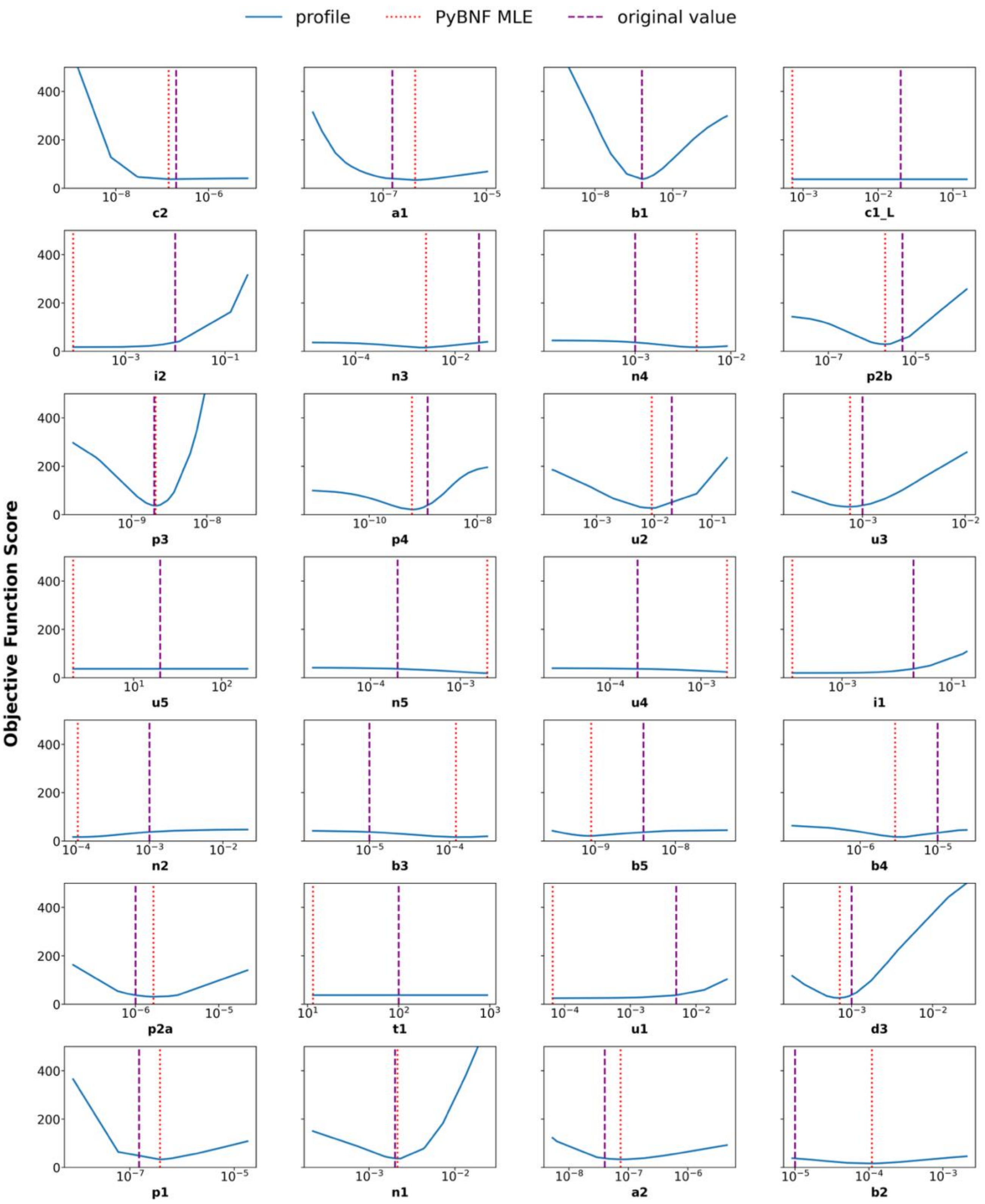

Supplementary Figure 4: A profile likelihood table of all 28 parameters used for parameterization with PyBioNetFit. The x axis (log scale) for each plot shows what parameter value corresponds to which objective function score on the y axis. Vertical, red-dotted lines indicate the maximum likelihood estimate for that parameter. Vertical, purple-dotted lines indicate the original parameter

value used by Kocieniewski and Lipniacki (2013). Parameters that are identifiable should have curved, bell-shaped, lines (blue lines) that decrease in objective function score until they reach their maximum likelihood estimates (red-dotted lines). Parameters that are not identifiable appear flat or near flat.

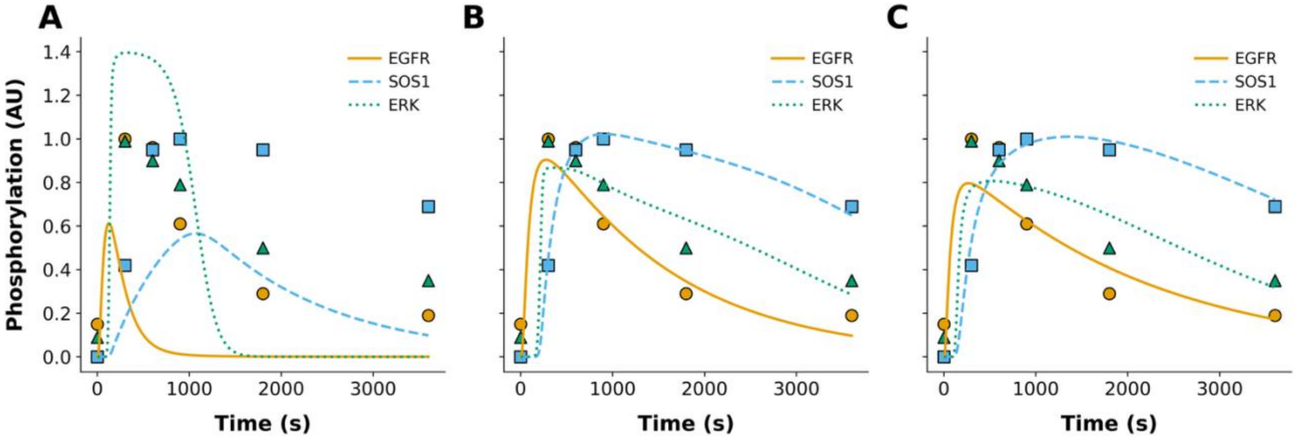

Supplementary Figure 5: Ablation analysis to assess the contributions of only BPSL constraints, only experimental data, and a combination of both data types to the overall fit to the experimental data. Panel A shows PyBioNetFit's overall fit to experimental data only using only BPSL statements (qualitative data) with an objective function value of **39**. Panel B shows PyBioNetFit's overall fit to the experimental data using only experimental data, with an objective function score of **8**. Panel C shows PyBioNetFit's overall fit to the experimental data using both BPSL statements (qualitative constraints) and experimental data with an objective function score of **24**. In all three instances, PyBioNetFit's differential evolution (DE) fitting algorithm was used along with PyBioNetFit's sum of squares (SOS) objective function to evaluate "closeness of fit". Lower objective function values indicate a closer fit between the projected time courses and the experimental data. Because there is experimental data available only to the Wild-Type model (WT), we could only use the WT-specific constraints (BPSL statements) to make a fair comparison between each plot. Thus, there were **6** WT BPSL statements (qualitative data) used in this ablation analysis. Using only BPSL statements (Panel A) PyBioNetFit's parameterization followed **6/6 constraints**. Using only experimental data (Panel B), PyBioNetFit's parameterization followed **6/6 constraints**. Using both BPSL statements and experimental data (Panel C), PyBioNetFit's parameterization followed **6/6 constraints**. These results demonstrate that incorporating both quantitative and qualitative constraints yields the most balanced and accurate parameterization, improving reproducibility without sacrificing fit quality.
